# Efficacy and Safety of Pharmacological and Psychological Interventions for the Treatment of Psychosis and Schizophrenia in Children, Adolescents and Young Adults: A Systematic Review and Meta-Analysis

**DOI:** 10.1371/journal.pone.0117166

**Published:** 2015-02-11

**Authors:** Megan R. Stafford, Evan Mayo-Wilson, Christina E. Loucas, Anthony James, Chris Hollis, Max Birchwood, Tim Kendall

**Affiliations:** 1 National Collaborating Centre for Mental Health (NCCMH), Royal College of Psychiatrists, London, United Kingdom; 2 Centre for Outcomes, Research & Effectiveness (CORE), University College London, London, United Kingdom; 3 Highfield Adolescent Unit, Warneford Hospital, Oxford, United Kingdom; 4 Faculty of Medicine & Health Sciences, Queen’s Medical Centre, Nottingham, United Kingdom; 5 Division of Mental Health and Wellbeing, Warwick Medical School, University of Warwick, Coventry, United Kingdom; Maastricht University, NETHERLANDS

## Abstract

**Background:**

Studies report contrasting results regarding the efficacy and safety of pharmacological, psychological, and combined interventions in psychosis and schizophrenia in children, adolescents and young adults.

**Methods:**

Systematic review and meta-analysis. Embase, Medline, PreMedline, PsycINFO, and CENTRAL were searched to July 2013 without restriction to publication status. Randomised trials comparing any pharmacological, psychological, or combined intervention for psychosis and schizophrenia in children, adolescents and young adults were included. Studies were assessed for bias, and GRADE criteria were used to describe the quality of the results.

**Results:**

Twenty-seven trials including 3067 participants were identified. Meta-analyses were performed for 12 comparisons: symptoms, relapse, global state, psychosocial functioning, depression, weight and discontinuation. Low quality evidence demonstrated that antipsychotics have small beneficial effects on psychotic symptoms (SMD = -0.42, 95% CI -0.58 to -0.26), and a medium adverse effect on weight gain (WMD = 1.61, 95% CI 0.61 to 2.60) and discontinuation due to side effects (RR = 2.44, 95% CI, 1.12 to 5.31). There were no trials of psychological treatments in under-18 year olds. There was no evidence of an effect of psychological interventions on psychotic symptoms in an acute episode, or relapse rate, but low quality evidence of a large effect for family plus individual CBT on the number of days to relapse (WMD = 32.25, 95% CI -36.52 to -27.98).

**Conclusions:**

For children, adolescents and young adults, the balance of risk and benefit of antipsychotics appears less favourable than in adults. Research is needed to establish the potential for psychological treatments, alone and in combination with antipsychotics, in this population.

## Introduction

Early-onset schizophrenia, that is, schizophrenia occurring prior to 17 years [1], affects approximately 1.6 to 1.9 per 100,000 of the child and adolescent population [2–5]. It is a severe and debilitating disorder associated with considerable long-term impairments in psychological, social, educational and occupational functioning [6], poor physical health, reduced life expectancy [7,8], and substantial direct and indirect costs [9,10]).

Compared with adult-onset schizophrenia, early-onset schizophrenia may be a more severe disorder, negatively influencing social, cognitive and psychological development [6]. While antipsychotic medications play an integral role in the treatment and management of schizophrenia in children, adolescents and young adults, the nature of adverse effects that can follow first exposure occurs during a vulnerable phase of physical growth and brain development, and at a time when young people may be particularly vulnerable to rapid weight gain [11] and disturbances to the cardiometabolic system [12,13], bone growth [14] and sexual development [15]. Such health risks raise important public health concerns given the widespread use of these medications [16]. Furthermore, children, adolescents and young adults are more likely than adults to exhibit negative symptoms, and less likely to exhibit systematized delusions and hallucinations [17]. This has implications for the potential efficacy in children, adolescents and young adults of psychological interventions developed for adults with psychosis or schizophrenia. The increased recognition of the limitations associated with antipsychotic medication has stimulated greater interest in psychological interventions in this population [18]. A recent systematic review of interventions for people who do not have established psychosis, found that psychological interventions may have a positive impact if delivered before the onset of psychosis in individuals with attenuated or transient psychotic symptoms [19]. Additionally, demand for psychological therapies in general has also grown. In England, this has culminated in the Department of Health’s Improving Access to Psychological Therapies (IAPT) initiative, which is set to receive further funding to extend to children, adolescent and young adults and to those with major mental health problems, particularly schizophrenia, under the UK coalition government’s mental health strategy [20]. Finally, families may play an even greater role in providing care and support to children, adolescents and young adults with schizophrenia compared to adults. Given the robust evidence for the efficacy of family interventions in adult schizophrenia [21], these interventions may be particularly promising in children, adolescents and young adults.

A previous review of antipsychotic medications for childhood-onset schizophrenia found limited evidence regarding the effectiveness of antipsychotic medication in this population [22], but searches were conducted in 2007 and the review did not include participants over the age of 13 years. The evidence indicates there are few advantages of second-generation antipsychotics over first-generation antipsychotics in treating psychosis [22], suggesting they could be combined in a meta-analysis. Research in this field has advanced rapidly in recent years, and a current review is needed to determine the efficacy and safety of pharmacological, psychological and combination interventions in the treatment of children, adolescents and young adults with psychosis and schizophrenia.

## Methods

This systematic review and meta-analysis was conducted as part of a clinical guideline for the management of psychosis and schizophrenia in children, adolescents and young adults [23], following a published protocol (see Appendix A in S1 File).

### Eligibility criteria

We included all randomised controlled trials evaluating pharmacological, psychological or combination treatment for children, adolescents and young adults (18 years of age or younger) with a first episode psychosis (FEP) (a first experience of psychotic symptoms [24]) or a subsequent acute episode of psychosis or schizophrenia. Given the evidence from longitudinal neuroimaging studies demonstrating that brain growth continues into the twenties [25], and epidemiological studies which show the incidence of schizophrenia rising in late adolescence and early adulthood [26], we included studies in which the sample consisted of some participants under 18 years and some over 18 years, as long as the sample mean age did not exceed 25 years. We excluded studies of individuals with bipolar disorder only; studies of people who failed to respond to previous antipsychotic medication (i.e. treatment resistant); studies comparing a single treatment without a placebo arm; studies containing less than 10 participants per group; and studies not available in English. In addition, the current systematic review and meta-analysis was conducted as part of a clinical guideline for England and Wales and therefore studies of drugs not licensed in the UK were also excluded.

### Types of outcome measures

Primary

We examined symptoms of psychosis (total, positive and negative) and relapse, at post-treatment and follow-up.

Secondary

We also analysed symptoms of depression, symptoms of anxiety, psychosocial functioning, global state, weight, and discontinuation due to side effects or for any reason.

### Search Strategy

We searched Embase, MEDLINE, PreMedline, PsycINFO and CENTRAL from inception to July 2013 (see Appendix B in S1 File for the Medline population terms and the full list of search terms used across databases). In addition, we searched the reference lists of included studies, excluded studies, and previous reviews, and contacted study authors and experts.

### Assessment of Bias

Studies were assessed independently by two authors (MRS, CEL) using the Cochrane Collaboration Risk of Bias Tool [27]. Disagreements were discussed with a third author (EMW) and resolved by consensus. Each study was rated for risk of bias due to: sequence generation; allocation concealment; blinding of participants, assessors, and providers; selective outcome reporting; and incomplete data. Risk of bias for each domain was rated as high (seriously weakens confidence in the results), low (unlikely to seriously alter the results) or unclear.

Due to the small number of trials, we were unable to assess publication bias formally (e.g. using a trim and fill analysis) [28]. However, previous reviews have demonstrated systematic under-reporting of such interventions in mental health for children, resulting in effects that have been systematically overstated and harms that have been systematically underestimated [29]. Many of these interventions were developed before the introduction of mandatory trial registration [30], rules with which manufacturers often fail to comply [31]. We believe there is a high risk of publication bias because the addition of one or two small unpublished studies could change our view of the relative benefits and harms of these interventions.

### Data Management

For continuous outcomes, the magnitude of treatment effects were calculated as a standardised mean difference (SMD), Hedges *g* [32]. A small effect was considered to be a SMD of between 0.00 and 0.49, a medium effect between 0.50 and 0.79 and a large effect >0.80. We also calculated the weighted mean difference (WMD) for weight and time to relapse when studies reported the same measure. Sensitivity analysis was conducted for continuous outcomes when endpoint and change data were included in the same analysis. For dichotomous outcomes, an overall risk ratio (RR) was calculated. We analysed individually randomised units. When data were extracted in several formats that could not be combined directly in RevMan, we used the generic inverse variance option. All outcomes are reported with 95% confidence intervals (CI). Overall effects were calculated using random-effects. Continuous effects were weighted by the inverse of variance; dichotomous effects were weighted using the Mantel-Haenszel method [33,34].

Missing data were noted for each outcome. When dropout was not reported, we contacted the authors. For both primary and secondary outcomes reporting data for completers as well as controlling for dropout (for example, imputed using regression methods), we used the latter.

Subgroup analyses were conducted for different doses of antipsychotic medication, where more than one dose was compared with placebo. We used the lower and upper dose ranges identified by the Prescribing Observatory for Mental Health, United Kingdom Topic 10 benchmarking exercise of antipsychotic prescribing in children and young people in practice [35], to categorise doses administered in the included trials as either ‘lower’ or ‘higher’ doses of medication. Therefore, ‘higher’ doses are those exceeding the maximum dose stated in the manufacturers’ summary of product characteristics for that drug, and ‘lower’ doses are those under the minimum dose stated in the manufacturers’ summary of product characteristics for that drug. Because children, adolescents and young adults previously unexposed to antipsychotics may be particularly vulnerable to weight gain associated with antipsychotic use [36], we also conducted subgroup analyses for FEP and subsequent acute episode groups. FEP and subsequent acute episode groups were defined as reported by the trial authors.

Statistical heterogeneity was assessed by visual inspection of forest plots, by performing the Chi^2^ test (assessing the p value), and by calculating the I^2^ statistic [37,38], which describes the percentage of observed heterogeneity that would *not* be expected by chance. If the p value was less than 0.10 and I^2^ exceeded 50%, we considered heterogeneity to be substantial. Meta-analyses were conducted using RevMan [39]. Confidence in the results was assessed using the GRADE method [40], which is a structured assessment of the quality of evidence attending to the following factors: (1) risk of bias; (2) inconsistency; (3) indirectness; (4) imprecision; and (5) publication bias.

## Results

### Trial flow

**Fig 1:**
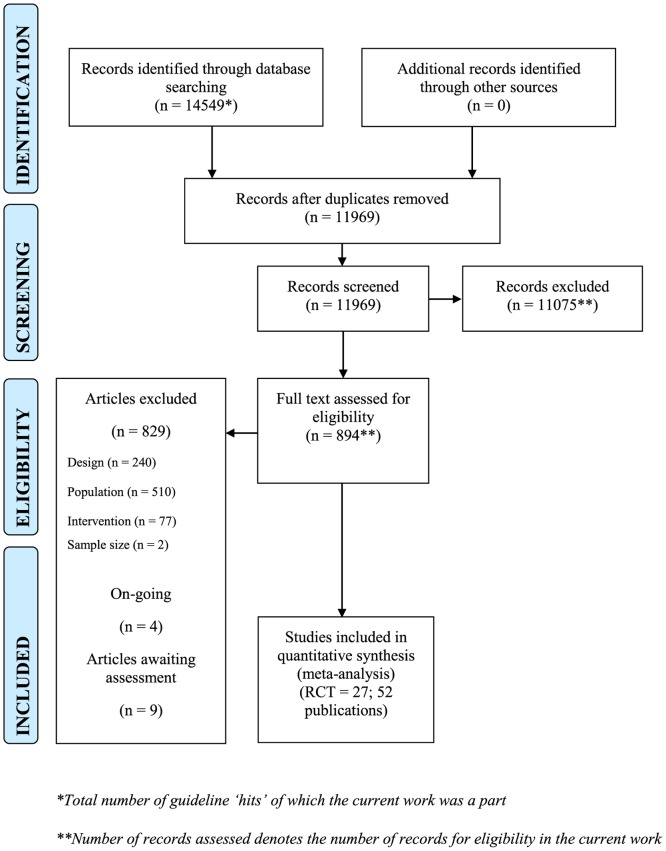
PRISMA flowchart.

This review was conducted as part of a clinical guideline for the management of psychosis and schizophrenia in children, adolescents and young adults which identified 11969 potentially relevant citations, for which 894 papers were retrieved. Of these, 829 were not relevant and were excluded. The most common reason for exclusion was that the study was conducted in an adult population and included no-one under the age of 18 years. Thirty studies were excluded from this review with reasons (Appendix C in S1 File). Two studies [41,42] were not published in English and were identified via an included systematic review of antipsychotic medication for childhood-onset schizophrenia [22]. Nine trials await assessment (six trials were not published in English, and three trials reported insufficient information in a conference abstract to make an assessment, see Appendix D in S1 File) and four ongoing trials were identified (Appendix E in S1 File). Therefore, 27 randomised controlled trials reported in 52 published papers were included in this systematic review and meta-analysis (Fig. 1).

### Study characteristics

Pharmacological study characteristics

Nineteen included pharmacological trials assigned 2338 participants. The median sample size was 75 (range 22 to 400) and 1552 (66%) randomised participants were male. Table 1 contains the characteristics of included pharmacological trials. Comparisons included seven placebo controlled trials and 12 head-to-head trials. The median length of treatment was 8 weeks (range 4 to 52) with only two trials reporting long-term follow-up assessments at 104 [43] and 156 weeks [44].

**Table 1:** Study characteristics for pharmacological interventions.

Study ID	N	Country	Mean age yrs (SD)	Intervention (mg/day)	PT (FU) weeks
*First episode psychosis*					
ARANGO2009	50	ESP	15.9 (1.3)	Quetiapine (438.8) vs olanzapine (12.1)	26 (None)
LIEBERMAN2003	263	Multiple	23.8 (4.8)	Olanzapine (10.2) vs haloperidol(4.82)	12 (104)
MCEVOY2007	400	Multiple	24.5 (5.8)	Olanzapine(11.7) vs quetiapine(506) vs risperidone (2.4)	52 (None)
ROBINSON2006	120	USA	23.3 (5.1)	Olanzapine (11.8) vs risperidone (3.9)	16 (156)
SIKICH2008b^1^	119	USA	13.8 (2.4)	Olanzapine (11.4) vs risperidone (2.8)	52^2^ (None)
SWADI2010	22	N Z	16.7 (nr)	Quetiapine (607.0) vs risperidone (2.9)	6 (None)
VANBRUGGEN2003	44	NL	20.8 (2.9)	Olanzapine (15.6) vs risperidone(4.4)	6–10 (None)
*Subsequent acute episode*					
FINDLING2012	222	Multiple	15.4 (1.3)	Quetiapine (400.0) vs quetiapine (800.0) vs placebo (na)	26 (None)
FINDLING2008A	302	Multiple	15.5 (1.4)	Aripiprazole (10.0) vs aripiprazole (30.0) vs placebo (na)	6 (None)
HAAS2009B	160	Multiple	15.6 (1.3)	Risperidone (1.0–3.0) vs risperidone (4.0–6.0) vs placebo (na)	6 (None)
JENSEN2008	30	USA	15.2 (2.1)	Olanzapine (14.0) vs quetiapine (611.0) vs risperidone (3.4)	12 (None)
KRYZHANOVSKAYA2009B	107	Multiple	16.7 (1.4)	Olanzapine (11.1) vs placebo (na)	6 (None)
MOZES2006	25	IL	11.1 (1.6)	Olanzapine (8.2) vs risperidone (1.6)	12 (None)
PAILLERE-MARTINOT1995	27	FR	20 (4.0)	Amisulpride (50.0–100.0) vs placebo (na)	6 (None)
POOL1976*	75^3^	USA	15.5 (nr)	Haloperidol (11.9) vs placebo (na)	4 (None)
SIKICH2004	51	USA	14.8 (2.8)	Olanzapine (12.3) vs risperidone (4.0) vs haloperidol (5.0)	8 (None)
SINGH2011^4^	201	Multiple	15.4 (1.5)	Paliperidone (1.5) vs paliperidone (3–6) vs placebo (na)	6 (None)
KENNEDY2012/ XIONG2003	60	CN	13.0 (nr)	Risperidone (0.5–5.0) vs chlorpromazine (50.0–400.0)	8 (None)
KENNEDY2012/ YAO2004	60	CN	11.0 (nr)	Risperidone (0.25–3.0) vs haloperidol (0.5–12.0)	6 (None)

*Note*.

* Data not reported in sufficient detail to include in analysis

^1^ Molindone was the third arm of this trial (n = 40), however as it was discontinued by its sole supplier, Endo Pharmaceuticals, on January 13, 2010, only data for risperidone and olanzapine are used in this review

^2^ The study design consisted of an 8 week ‘acute phase’ and a blind ‘maintenance phase’ up to 52 weeks post randomization. During the maintenance phase participants continued to be administered treatment within their randomised groups and at same dose range.

^3^ Loxapine was the third arm of this trial (n = 26), however it was not included in this guideline as it was discontinued in the UK in 2003.

^4^This trial included a fourth arm of paliperidone 6–12mg/day. The 3–6mg/day arm was selected as the ‘higher dose’ antipsychotic medication in accordance with POMH-UK Topic 10 benchmarking exercise and therefore the 6–12mg/day arm was not included in the current work.

N = number randomised; nr = not reported; na = not applicable; mg = milligrams; DU = duration of treatment; PT = post-treatment data collection; FU = follow-up data collection

We conducted meta-analysis for seven pharmacological comparisons. Antipsychotic drugs that were compared with a placebo included quetiapine [45], aripiprazole [46], risperidone [47], paliperidone [48], amisulpride [49], olanzapine [50] and haloperidol [51]. The median of the mean ages was 15.5 years (range 15.4 to 20.0 years). None of these trials were conducted in FEP. These trials were included in a meta-analysis of antipsychotic medications compared to placebo and subgroup analyses were conducted for ‘lower’ and ‘higher’ doses of antipsychotic medication (see ‘Data management‘). Total, positive and negative symptoms were measured using the Positive and Negative Syndrome Scale (PANSS) and global state was measured using the Clinical Global Impressions Scale (CGI). A variety of measures were used to measure depression including the Montgomery-Åsberg Depression Rating Scale (MADRS), the Hamilton Depression Rating Scale (HAM-D) and the PANSS-Depression; and psychosocial functioning including the Children’s Global Assessment Scale (CGAS) and the Global Assessment of Functioning (GAF). Relapse and anxiety were not measured in any trials of antipsychotic medication compared with placebo.

Additionally, head-to-head comparisons included risperidone compared with olanzapine [44,52–56], haloperidol [22,53], quetiapine [55,57,58], and chlorpromazine [22]; and olanzapine compared with quetiapine [55,57,59] and haloperidol [43,53]. The median of the mean ages was 15.5 years (range 11.0 to 24.5 years). Subgroup analyses were conducted for FEP and subsequent acute episode groups. Total, positive and negative symptoms were measured using the Positive and Negative Syndrome Scale (PANSS) and the Brief Psychiatric Rating Scale (BPRS). Global state and psychosocial functioning were measured using the CGI and the CAS respectively. Relapse and anxiety were not measured in any of the included head-to-head trials.

Psychological study characteristics

Eight included psychological trials assigned 729 participants with a median sample size of 64 (range 30 to 309). The median of the mean ages was 22.3 years (range 15.0 to 24.0 years), with no samples of exclusively under 18 year olds identified. Four hundred and fifty seven (63%) randomised participants were male. The median length of treatment across trials was 22 weeks (range 10 to 65) and all trials except one [60] conducted follow-up assessments, with a median follow-up of 78 weeks (range 26 to 260). All participants were either currently experiencing FEP or a subsequent acute episode of psychosis or schizophrenia, apart from two trials that were specifically designed to test a relapse prevention strategy following remission from first psychotic episode [61,62]. Table 2 contains the characteristics of included psychological trials.

**Table 2:** Study characteristics for psychological interventions.

Study ID	N	Country	Mean age yrs (SD)	Intervention (mg/day)	PT (FU) weeks
APTER1978*	30	NR	nr	Individual movement therapy vs group movement therapy vs non-specific dance therapy	12 (None)
JACKSON2008	62	AU	22.3 (3.6)	Individual CBT + EPPIC TAU vs befriending + EPPIC TAU	14 (52)
JACKSON2009	66	AU	23.3 (4.6)	Individual CBT vs TAU	26 (52)
HADDOCK2006*	309	GBR	nr	Individual CBT+TAU (UK) vs supportive counselling+TAU (UK) vs TAU	18 (78)
MAK2007*	48	CN	24 (4)	Individual CBT vs waitlist	CBT:36 (60); waitlist:26 (84)
POWER2003	56	AU	nr (range 15–29)	Individual CBT + EPPIC TAU in acutely suicidal patients vs EPPIC TAU in acutely suicidal patients	10 (26)
GLEESON2009	82	AU	20.1 (3.1)	Family CBT + individual CBT vs EPPIC TAU	28 (130)
LINSZEN1996	76	NL	20.6 (2.5)	Family CBT vs individual CBT	65 (260)

*Note*.

* Data not reported in sufficient detail to include in analysis

CBT = cognitive behavioural therapy; EPPIC = Early Psychosis Prevention and Intervention Centre; TAU = treatment as usual; UK = United Kingdom; N = number randomised; nr = not reported; na = not applicable; PT = post-treatment data collection; FU = follow-up data collection

We conducted meta-analysis for five psychological comparisons. Psychological interventions included arts therapy, CBT and family interventions. Comparisons included individual body movement therapy compared with group body movement therapy and a non-specific dance therapy control [60]; CBT compared with waitlist [63]; CBT compared with treatment as usual (TAU) [64,65]; CBT compared with supportive counselling [64]; CBT compared with befriending [66]; CBT for acutely suicidal patients compared with TAU [67]; family CBT compared with individual CBT [61]; and family plus individual CBT compared with TAU [62]. Three of eight included trials were conducted in a specialist Early Psychosis Prevention and Intervention Centre (EPPIC) in Australia, which offers a highly comprehensive service to people aged 15 to 25 years with emerging psychotic disorders [62,66,67]. All participants in these studies received TAU by the EPPIC centre. Appendix F in S1 File provides a detailed description of the psychological interventions used in the included trials. Total, positive and negative symptoms were measured using the Positive and Negative Syndrome Scale (PANSS) and the Brief Psychiatric Rating Scale (BPRS). The Scale for the Assessment of Negative Symptoms (SANS) was also used to measure negative symptoms. Relapse was measured using the BPRS. Depression was measured using the Beck Depression Inventory (BDI), Montgomery-Åsberg Depression Rating Scale (MADRS), and the Calgary Depression Scale for Schizophrenia (CDSS). Global state was measured using the Clinical Global Impressions scale (CGI). Psychosocial functioning and anxiety were not measured in any of the psychological trials.

### Risk of bias

We rated risk of bias for each trial (Appendix G in S1 File) using the Cochrane risk of bias tool [27].

Pharmacological trials

Nine out of 19 included pharmacological trials employed adequate methods of sequence generation, however the risk of bias due to inadequate allocation concealment was unclear in 17 trials. Lack of blinding of assessors created a high risk of bias for some outcomes in two studies and for 12 studies this was unclear. Five studies were at high risk of bias because participants or staff were not blind and for 11 studies this was unclear. There was a high risk of bias due to incomplete outcome data for 15 included trials and only two trials were clearly free of attrition bias. Only nine studies were clearly free of selective outcome reporting; seven trials did not report all outcomes and for two trials data were not reported in sufficient detail to be included in meta-analysis. It was unclear whether three trials reported all outcomes.

Psychological trials

Four out of eight included psychological trials were considered to have employed adequate methods of sequence generation, however the risk of bias owing to poor allocation concealment was unclear for all trials. There was a high risk of bias due to incomplete outcome data for four trials, and for one trial this was unclear. Four trials were at a high risk of selective outcome reporting, and for three of these trials, no data could be extracted for any outcomes.

### Quantitative data synthesis

We analysed psychotic symptoms, relapse, global state, psychosocial functioning, depression, weight and discontinuation of treatment. Summary of effects for all comparisons and all outcomes can be found in Appendix H in S1 File.

Pharmacological quantitative data synthesis

A meta-analysis of antipsychotic medications compared to placebo was conducted because of the small number of trials and participants, and therefore lack of statistical power, for any single antipsychotic compared to placebo. Six of seven placebo-controlled trials reported data for at least one outcome in sufficient detail to be included in this meta-analysis [45–50]. Subgroup analyses were conducted for ‘lower’ and ‘higher’ doses of antipsychotic medication.

Eleven of twelve head-to-head trials reported data for at least one outcome to be included in meta-analysis [22,43,52–59]. We analysed nine trials comparing two antipsychotics and three trials comparing three antipsychotics. Subgroup analyses were conducted for FEP and subsequent acute episodes of schizophrenia.

Efficacy of antipsychotic medication versus placebo

**Fig 2:**
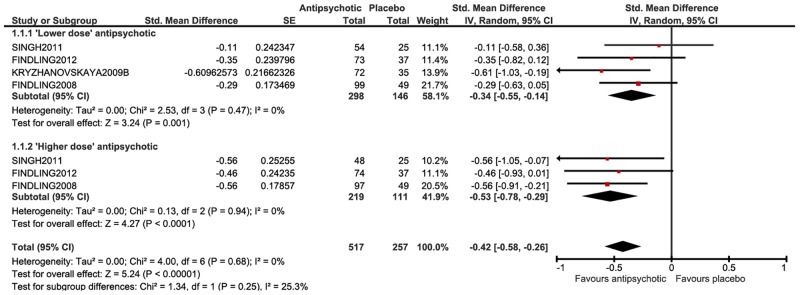
Antipsychotic medication compared with placebo at post-treatment—total symptoms.

At post-treatment, low quality evidence suggested small effects for antipsychotic medication on total symptoms (SMD = -0.42, 95% CI-0.58 to-0.26) (Fig. 2), positive symptoms (SMD = -0.42, 95% CI-0.56 to-0.28), negative symptoms (SMD = -0.32, 95% CI-0.46 to-0.18), depression (SMD = -0.24, 95% CI-0.45 to-0.03), psychosocial functioning (SMD = -0.37, 95% CI-0.52 to-0.23) and global state severity (SMD = -0.41, 95% CI-0.58 to-0.25), and a large effect for global state improvement (RR **=** 1.89, CI 1.26 to 2.83). These effects remained small but statistically significant in subgroup analyses, with marginal decreases and increases in the size of the effects for ‘lower dose’ and ‘higher dose’ groups respectively. However, the effect of treatment was relatively small compared with change over time, even in the absence of intervention. In one trial, PANSS scores fell approximately 20 points in the placebo group [45], constituting a minimum clinically important difference of greater than 15 PANSS points as estimated by Hermes et al. [68]. The difference between post-treatment mean scores between groups was approximately eight points, and in another trial, the difference between placebo and antipsychotic treated groups was approximately nine points [48]. These differences are small and do not meet this threshold.

Side effects of antipsychotic medication versus placebo

**Fig 3:**
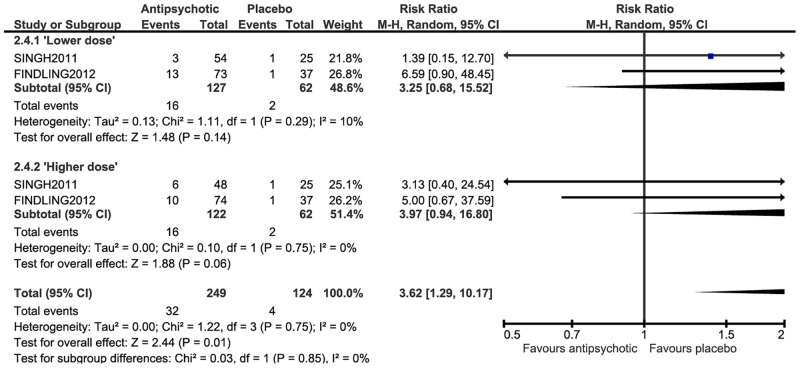
Antipsychotic medication compared with placebo at post-treatment—weight.

There was very low quality evidence for a medium effect on weight (kg), with antipsychotic treated participants gaining significantly more weight than the placebo group at post-treatment (SMD = 0.63, 95% CI 0.32 to 0.93) (WMD = 1.61, 95% CI 0.61 to 2.60), however there was significant heterogeneity across studies (p = 0.0007, I^2^ = 68% and p = 0.00001, I^2^ = 84% respectively). A large effect was observed for the number of participants gaining >7% of their baseline body weight (RR = 3.62, 95% CI 1.29 to 10.17) (Fig. 3), however, there was no significant effect for either of the ‘lower’ and ‘higher’ dose subgroups (‘lower dose’: RR = 3.25, 95% CI 0.68 to 15.52; ‘higher dose’: RR = 3.97, 95% CI 0.94 to 16.80). A large effect favouring placebo was found for leaving the study early due to side effects at post-treatment (RR = 2.44, 95% CI, 1.12 to 5.31), however, there was no significant effect for either of the ‘lower’ and ‘higher’ dose subgroups (‘lower dose’: RR = 2.53, 95% CI 0.87 to 7.34; ‘higher dose’: RR = 2.33, 95% CI 0.74 to 7.30).

Efficacy of antipsychotic medication in head-to-head trials

In very low quality evidence there were no significant effects between antipsychotics in head-to-head trials for any of our included measures of efficacy, except a small effect for olanzapine on negative symptoms compared with haloperidol in FEP participants (SMD = -0.25, 95% CI-0.50 to-0.00), and a small effect for risperidone compared with quetiapine on positive symptoms in FEP participants (SMD = -0.43, 95% CI-0.82 to-0.03). Relapse and anxiety were not measured in any of the included head-to-head trials. Psychosocial functioning was not measured in trials of olanzapine compared with haloperidol, and depression was not measured in trials of risperidone compared with haloperidol.

Side effects of antipsychotic medication in head-to-head trials

For weight gain in FEP participants, very low quality evidence indicated a large differential effect favouring quetiapine to olanzapine (RR = 1.86, 95% CI 1.33 to 2.61), and moderate differential effects, favouring risperidone to olanzapine (RR = 0.68, 95% CI 0.47 to 0.98), and on haloperidol to olanzapine (SMD = 0.70, 95% CI 0.45 to 0.95) (WMD = 6.08, 95% CI 3.97 to 8.20). A large effect was observed in the FEP subgroup, favouring olanzapine to haloperidol, for leaving the study early due to side effects at post-treatment (RR = 0.37, 95% CI 0.16 to 0.85). In very low quality evidence there were no further significant effects on weight or leaving the study early due to side effects, between antipsychotics in head-to-head trials.

Psychological quantitative data synthesis

Five of eight included psychological trials reported data for at least one outcome in sufficient detail to be included in an analysis [61,62,65–67], however the comparators used in these trials were considered to be too different to combine in a meta-analysis and so single pairwise comparisons were conducted.

Family and individual CBT compared to TAU at the Early Psychosis Prevention and Intervention Centre

**Fig 4:**
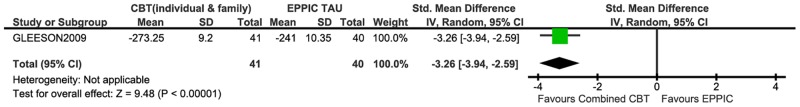
Family plus individual CBT compared with TAU at EPPIC—Time to relapse at post-treatment.

At 33 weeks post-treatment, there was low quality evidence that time to relapse was significantly extended by 32.25 days in family plus individual CBT compared to TAU at EPPIC (SMD = -3.26, 95% CI-3.94 to-2.59) (WMD = 32.25, 95% CI-36.52 to-27.98) (Fig. 4) [62]. Time to relapse was not reported at 130 weeks follow-up. However, the number of participants relapsing was not significantly different between groups at 33 weeks post-treatment (RR = 0.24, 95% CI 0.06 to 1.08) or at 130 weeks follow-up (RR = 0.98, 95% CI 0.31 to 3.11). No differential effects between groups were found for total or positive symptoms at either time point, or for negative symptoms at 33 weeks post-treatment, however a medium effect favouring TAU at EPPIC was found for negative symptoms at 130 weeks follow-up (SMD = 0.60, 95% CI 0.15 to 1.05). No significant difference was found between groups on psychosocial functioning at 33 weeks post-treatment, however a small effect for psychosocial functioning was observed at 130 weeks follow-up (SMD = -0.45, 95% CI-0.89 to-0.01). No differential effects between groups were found for depression or leaving the study early for any reason. Anxiety and global state were not measured in this trial.

CBT compared with TAU in the UK

No significant differences were found for depression between CBT and TAU in the UK at post treatment (SMD = -0.29, 95%CI-0.87 to 0.30) or follow-up (SMD = -0.05, 95% CI-0.63 to 0.52); or leaving the study early for any reason at post-treatment (RR = 1.94, 95% CI 0.85 to 4.43) or follow-up (RR = 1.77, 95% CI 0.89 to 3.52) [65]. Symptoms, relapse, global state, psychosocial functioning and anxiety were not measured in this trial.

CBT plus TAU compared with befriending at EPPIC

No significant differences were found between CBT plus TAU at EPPIC and befriending at post treatment for positive symptoms (SMD = -0.05, 95% CI-0.55 to 0.45), negative symptoms (SMD = -0.44, 95% CI-.095 to 0.06), and psychosocial functioning (SMD = -0.40, 95% CI-0.90 to 0.11) [64]. These effects remained non-significant at follow-up: SMD = -0.08, 95% CI-0.58 to 0.42 (positive symptoms), SMD = -0.37, 95% CI-0.87 to 0.13 (negative symptoms) and SMD = -0.08, 95% CI-0.58 to 0.41 (psychosocial functioning). No significant differences were found between CBT plus TAU at EPPIC and befriending on leaving the study early for any reason (RR = 0.57, 95% CI 0.19 to 1.76). Relapse, depression, global state and anxiety were not measured by this trial.

CBT compared with TAU for acutely suicidal patients at EPPIC

In a trial conducted in acutely suicidal participants, no significant differences were found between CBT and TAU at EPPIC for leaving the study early at post-treatment (RR = 2.02, 95% CI 0.72 to 5.66) [67]. The primary outcome in this trial was suicidality; however symptom and global state data were also collected, but not reported in sufficient detail to be included in an analysis. Relapse, depression, anxiety and psychosocial functioning were not measured in this trial.

Family CBT compared with individual CBT

No significant differences were found between family CBT and individual CBT at post-treatment for rates of relapse (RR = 0.95, 95% CI 0.34 to 2.68), and symptom data was collected but not reported in sufficient detail to be used in an analysis [61]. Global state, psychosocial functioning, depression, anxiety and numbers of participants leaving the study early for any reason were not measured by this trial.

## Discussion

### Findings

This is the first systematic review and meta-analysis of pharmacological and psychological treatments for children, adolescents and young adults with psychosis and schizophrenia. The data set on antipsychotics includes 19 trials, with 2338 participants with a median mean age of 15.5 years (range 11.0 to 24.5 years). Low quality evidence suggests small effects for antipsychotic medication on positive and negative symptoms, depression and psychosocial functioning and a large effect on global state, but also a medium effect on weight gain which increased quickly, especially over the first six weeks of treatment, with a median of the mean change in weight across antipsychotic treated participants of 1.25kg (range 0 to 4.3kg); and greater discontinuation due to side effects where this was reported. We note that in these trials the placebo groups improved substantially, with antipsychotics adding relatively small additional benefit. Head-to-head trials of antipsychotics showed medium to large differential effects on weight gain with olanzapine. However, most head-to-head trials of antipsychotics were underpowered and the evidence was very low quality, making any comparisons between individual antipsychotics unreliable.

The data set for psychological treatments amounted to eight trials, including 729 participants. Disappointingly, there were no psychological treatment trials for children or young people under 18 years of age reporting data in a sufficient format to be included in an analysis; but for young people under 25 years old, we found some low quality evidence that combining family interventions with individual CBT had a strong, statistically significant effect on extending time to relapse, although there were no differences in relapse rate. No significant beneficial effects were found for psychological interventions for psychotic symptoms or for depression. It was not possible to meta-analyse data from any of the psychological treatment trials, largely because of variation in controls, and we found no other statistically significant effects.

It is important to note, however, that in several psychological treatment trials, the control conditions included active interventions which mirror the effect of the psychological interventions in practice, where they are always administered in addition to treatment as usual. For example, three studies [62,66,67] were conducted in EPPIC, a very intensive and comprehensive treatment centre which includes an inpatient unit, an outpatient case management system, family work, accommodation, prolonged recovery programmes, tailored group programmes and clear, low dose medication protocols.

### Limitations

The two most important limitations we identified for this review were the age range of participants in studies included and the quality of the evidence. The dataset for children, adolescents and young adults under 18 years old is very much smaller than in adults (k = 10; n = 1217), with no studies in under 18 year olds identified for psychological interventions. Furthermore, most studies used outcomes measures that have been validated in adults. In addition, the quality of the evidence across the whole dataset was poor.

The quality of the evidence for antipsychotics was low or very low. Most trials were at high or unclear risk of selection bias and some trials were rated as having high or unclear performance and detection bias. However, we considered it unlikely that blinding of participants or providers would introduce any important bias, and we did not downgrade for this reason. Only two trials were rated as having low risk of attrition bias and less than half of the trials were completely free of selective outcome reporting, with many studies not reporting all outcomes. Two trials could not be included in any analyses because data reporting was inadequate. Another important reason for downgrading the quality of the evidence is the high risk of selective publication bias. The small number of trials meant it was not possible to assess publication bias formally (e.g. using a trim and fill analysis [28]).

Another limitation is the range of available outcomes, in particular relating to side effects. Weight was the most consistently reported outcome across trials, while other potentially relevant outcomes of antipsychotics, such as extrapyramidal side effects and other metabolic changes, are reported far less frequently. Poor reporting of side effects also raises the possibility of selective publication of outcomes and of whole studies, a practice that is common and leads to overestimating the benefits and underestimating the harm of drug treatments [29]. Unfortunately, it is not possible to be sure if all negative trials have been published.

The quality of the evidence for the psychological treatment trials was also low or very low. Several studies were at high risk of selection bias, attrition bias and selective outcome reporting. There was also a high risk of performance bias, but a low risk of detection bias across trials. As with pharmacological trials, we did not downgrade outcomes due to the risk of performance bias. Reassuringly, we found no evidence of psychological treatment trials being registered but not published.

### Conclusion

Compared to the substantially larger data-set for both pharmacological and psychological treatment trials for adults with psychosis and schizophrenia [69], this review suggests that while the efficacy of antipsychotics is similar in children, adolescents and young adults, side effects, in particular weight gain, are greater. The discontinuation rates due to side effects also suggests that these drugs are not well tolerated and that the balance of risks and benefits for antipsychotics may be less favourable in children, adolescents and young adults. This is in line with previous reviews of antipsychotics in children that conclude the benefits of antipsychotic medication are offset by the risks of serious side effects [22,70] and a cohort study demonstrating substantial weight gain following 12 weeks of treatment with antipsychotics [36]. The improvements observed for placebo treated groups in the current work points to a possible role of support and the passage of time, in recovery. A recent study shows that the difference between drugs and placebo becomes smaller as the length of the studies increase [71] and it remains to be tested whether the endpoint level of recovery remains different after a longer follow-up. The weight gain we have observed here, suggests that long, comprehensive investigations are needed for a complete and balanced cost-benefit analysis of long-term use of antipsychotic drugs, beginning in adolescence. In addition, the value of psychological treatments remains uncertain and largely untested in the young. Trials of psychological treatments in adults, strongly suggest that family interventions clearly and reliably reduce relapse rates and CBT reduces symptoms and length of hospitalisation [69]; with similar effects for people with first episode psychosis across all ages, both within and without early interventions services [21]. This could lead us to infer that these treatments are likely to be effective in younger age groups. However, this cannot be assumed for reasons such as cognitive immaturity. The lack of effect of psychological interventions on psychotic symptoms in our analyses suggests that similar benefits cannot be assumed when using interventions developed for adults with children, adolescents and young adults.

Psychosis and schizophrenia in children, adolescents and young adults are very serious and debilitating illnesses, which in clinical practice usually leads to the use of antipsychotics. However, in the absence of high quality evidence for the effectiveness of antipsychotic medication in children, adolescents and young adults, their routine use in the treatment of psychosis and schizophrenia should be undertaken cautiously. Furthermore, given the growing evidence that antipsychotics are associated with often severe metabolic, neurological and other side effects associated with significant premature mortality [72], these drugs should only be used under specialist psychiatric supervision and with careful monitoring [23]. Moreover, the complete absence of research on the effectiveness of psychological interventions in individuals under 18 years old needs to be urgently addressed. This is practically important because young people should not be denied potentially effective interventions because of an absence of research evidence. Although treatments developed for adults cannot be assumed to have the same benefits in children, adolescents and young adults, their benefits in first episode psychosis [21] suggests that family interventions and CBT would be good candidates for further research in younger age groups.

This review, we hope, can be used as a platform from which we can develop new clinical and research strategies, investigating pragmatic questions such as the benefits of combining family and individual psychological interventions, how psychological interventions should be adapted for children, adolescents and young adults, the benefits of psychological interventions alone and with limited and targeted use of antipsychotics, the predictors of response for different treatment approaches, and the most effective timing for interventions during the course of illness.

## Supporting Information

S1 File Appendix A, Review protocols. Appendix B, Search strategy. Appendix C, Excluded studies. Appendix D, Studies awaiting assessment. Appendix E, On-going studies. Appendix F, Description of included psychological and psychosocial interventions. Appendix G, Risk of bias assessments for included trials. Appendix H, Summary of effects. Appendix I, PRISMA checklist. (DOCX)
