# Supplementary material for: Efficacy and Safety of Pharmacological and Psychological Interventions for the Treatment of Psychosis and Schizophrenia in Children, Adolescents and Young Adults: A Systematic Review and Meta-Analysis
Source: PLoS One. 2015 Feb 11;10(2):e0117166. doi: 10.1371/journal.pone.0117166 (PMC4324833; doi:10.1371/journal.pone.0117166)
Supplement: S1 File — (DOCX) [file pone.0117166.s001.docx]

File SI: Supporting Information

Appendices

Appendix A: Review protocols

| Clinical review protocol for the review of initial treatment with antipsychotic medication in children and young people with first episode psychosis | |
| --- | --- |
| *Review questions* | **RQ B2:** Does the efficacy profile of continuous antipsychotic drug treatment, compared with alternative management strategies (placebo, another drug treatment, psychological interventions, psychosocial interventions) differ between children/young people and adults with psychosis and schizophrenia?  **RQ B3:** Are children and young people with psychosis and schizophrenia more susceptible to side effects of antipsychotic medication, compared with adults with psychosis and schizophrenia (in particular, metabolic, neurological and cognitive impairments)?  **RQ B5a:** Should the dose/duration (and, where relevant, frequency) be different compared with adults? |
| *Objectives* | To provide evidence-based recommendations regarding the pharmacological (antipsychotic) treatment and management of initial treatment in children and young people with psychosis and schizophrenia, including a review of the adult *Schizophrenia* guideline (NICE, 2009a) for its relevancy to children and young people. |
| *Population* | **Inclusion:** Children and young people (aged 18 years and younger) with first episode psychosis. Consideration will also be given to the specific needs of children and young people with psychosis and schizophrenia who have a mild learning disability and those from black and minority ethnic groups.  **Exclusion:** Study samples consisting only of individuals with a formal diagnosis of bipolar disorder. |
| *Intervention(s)* | All antipsychotic medication licensed in the UK for the treatment of children and young people with psychosis and schizophrenia, including considerations related to the age of participants (for example, dose modifications). Off-label use may be considered if clearly supported by evidence (for example, those licensed only for adults with psychosis and schizophrenia).   - Amisulpride - Aripiprazole - Benperidol - Chlorpromazine hydrochloride - Clozapine - Flupentixol - Haloperidol - Levomepromazine - Olanzapine - Pericyazine - Pimozide - Prochlorperazine - Promazine hydrochloride - Quetiapine - Risperidone - Sulpiride - Trifluoperazine - Zuclopenthixol - Zuclopenthixol acetate |
| *Comparison* | Alternative management strategies:   - Placebo - Psychological intervention   Any of the above interventions offered as an alternative management strategy. |
| *Critical outcomes* | - Mental state (symptoms, depression, anxiety, mania) - Mortality (including suicide) - Global state - Psychosocial functioning - Social functioning - Leaving the study early for any reason - Adverse effects (including effects on metabolism, EPS, hormonal changes and cardiotoxicity) - Remission |
| *Electronic databases* | **RQ B2 and RQ B5:**  Core databases: Embase, MEDLINE, MEDLINE In-Process, PsycINFO  Topic specific databases and grey literature (see Appendix 8)  **RQ B3:**  Core databases: Embase, MEDLINE, MEDLINE In-Process, PsycINFO  Topic specific databases (see Appendix 8) |
| *Date searched* | Systematic reviews: 1995 to May 2012  RCTs: inception of databases to May 2012 |
| *Study design* | RCTs; systematic reviews |
| *Review strategy* | - Two independent reviewers will review the full texts obtained through sifting all initial hits for their eligibility according to the inclusion criteria outlined in this protocol. - The initial approach is to conduct a meta-analysis evaluating the benefits and harms of pharmacological treatment. However, in the absence of adequate data, the literature will be presented via a narrative synthesis of the available evidence. - In order to assess the possible side effects of antipsychotic medication, children and young people with psychosis and schizophrenia will be included. In order to assess the efficacy of antipsychotic medication, children and young people with a formal diagnosis of schizophrenia will be included. - The main review will focus on children and young people between the ages of 14 and 18 years. The review will seek to identify whether modifications in treatment and management of children at or under 13 need to be made. Data from studies in which the study sample consists of children and young people under and over 18 years, but with a sample mean age of under 25, will be extrapolated if only limited evidence for children and young people aged 18 and younger is available. - Unpublished data will be included when the evidence is accompanied by a trial report containing sufficient detail to properly assess the quality of the data. The evidence must be submitted with the understanding that data from the study and a summary of the study’s characteristics will be published in the full guideline. Unpublished data will not be included when evidence submitted is commercial in confidence. |
| *Note.* ^1^ Off-label use may be considered if clearly supported by evidence (for example, those licensed only for adults). | |

| Clinical review protocol for the review of antipsychotics in the treatment of the acute episode in children and young people | |
| --- | --- |
| *Review questions* | **RQ B2**: Does the efficacy profile of continuous antipsychotic drug treatment, compared with alternative management strategies (placebo, another drug treatment, psychological interventions, psychosocial interventions) differ between children/young people and adults with psychosis and schizophrenia?  **RQ B3**: Are children and young people with psychosis and schizophrenia more susceptible to side effects of antipsychotic medication, compared with adults with psychosis and schizophrenia (in particular, metabolic, neurological and cognitive impairments)? |
| *Objectives* | To provide evidence-based recommendations regarding the pharmacological (antipsychotic) treatment and management of the acute episode in children and young people with psychosis and schizophrenia, including a review of the adult *Schizophrenia* guideline (NICE, 2009a) for its relevancy to children and young people. |
| *Population* | **Inclusion:** Children and young people (aged 18 years and younger) with an acute episode of psychosis and schizophrenia. Consideration will also be given to the specific needs of children and young people with psychosis and schizophrenia who have a mild learning disability and those from black and minority ethnic groups.  **Exclusion:** Study samples consisting only of individuals with a formal diagnosis of bipolar disorder. |
| Intervention(s) | All antipsychotic medication licensed in the UK for the treatment of children and young people with psychosis and schizophrenia, including considerations related to the age of participants (for example, dose modifications). Off-label use^1^ may be considered if clearly supported by evidence (for example, those licensed only for adults with psychosis and schizophrenia).   - Amisulpride - Aripiprazole - Benperidol - Chlorpromazine hydrochloride - Clozapine - Flupentixol - Haloperidol - Levomepromazine - Olanzapine - Pericyazine - Pimozide - Prochlorperazine - Promazine hydrochloride - Quetiapine - Risperidone - Sulpiride - Trifluoperazine - Zuclopenthixol - Zuclopenthixol acetate |
| *Comparison* | Alternative management strategies   - Placebo - Psychological intervention - Any of the above interventions offered as an alternative management strategy |
| *Critical outcomes* | - Mental state (symptoms, depression, anxiety, mania) - Mortality (including suicide) - Global state - Psychosocial functioning - Social functioning - Leaving the study early for any reason - Adverse effects (including effects on metabolism, EPS, hormonal changes, cardiotoxicity) - Remission |
| *Electronic databases* | **RQ B2**:  Core databases: Embase, MEDLINE, MEDLINE In-Process, PsycINFO  Topic specific databases: AEI, AMED, Applied Social Services Index and Abstracts (ASSIA), BEI, CDSR, CENTRAL, CINAHL, DARE, ERIC, HTA, IBSS, Sociological Abstracts, SSA, SSCI  Grey literature databases: HMIC, PsycBOOKS, PsycEXTRA  **RQ B3**:  Core databases: Embase, MEDLINE, MEDLINE In-Process, PsycINFO  Topic specific databases: CDSR, CENTRAL, DARE |
| *Date searched* | Systematic reviews: 1995 to May 2012  RCTs: inception of databases to May 2012 |
| *Study design* | Systematic review, RCT |
| *Review strategy* | - Two independent reviewers will review the full texts obtained through sifting all initial hits for their eligibility according to the inclusion criteria outlined in this protocol. - The initial approach is to conduct a meta-analysis evaluating the benefits and harms of pharmacological treatment. However, in the absence of adequate data, the literature will be presented via a narrative synthesis of the available evidence. - The main review will focus on children and young people between the ages of 14 and at or under 18 years. The review will seek to identify whether modifications in treatment and management of children aged at or under 13 yearsneed to be made. Data from studies in which the study sample consists of children and young people under and over 18 years, but with a sample mean age of under 25, will be extrapolated if only limited evidence for children and young people aged 18 and younger are available. - Unpublished data will be included when the evidence is accompanied by a trial report containing sufficient detail to properly assess the quality of the data. The evidence must be submitted with the understanding that data from the study and a summary of the study’s characteristics will be published in the full guideline. Unpublished data will not be included when evidence submitted is commercial in confidence. |
| *Note.* ^1^ Off-label use may be considered if clearly supported by evidence (for example, those licensed only for adults). | |

| Clinical review protocol for the review of psychological and psychosocial interventions for children and young people with psychosis and schizophrenia | |
| --- | --- |
| Component | Description |
| *Review question* | **RQ B11**: Do the advantages and disadvantages of psychological or psychosocial interventions, compared with alternative management, differ between children/young people and adults with psychosis and schizophrenia?  **RQ B12**: Are the advantages and disadvantages of combining particular psychological/ psychosocial interventions with an antipsychotic, either concurrently or sequentially, different for children and young people with psychosis and schizophrenia compared with adults with psychosis and schizophrenia?  **RQ B13:** Should the duration (and, where relevant, frequency) of an initial psychological/ psychosocial intervention be different in children and young people with psychosis and schizophrenia compared with adults with psychosis and schizophrenia?  **RQ B14**: Is the most effective format for particular psychological/ psychosocial interventions (for example, group or individual) the same for children and young people with psychosis and schizophrenia compared with adults with psychosis and schizophrenia? |
| *Objectives* | To provide evidence-based recommendations regarding the psychological and psychosocial treatment and management of children and young people with psychosis and schizophrenia, including a review of the adult *Schizophrenia* guidelinen (NICE, 2009a, NCCMH, 2010), for its relevance to children and young people. |
| *Population* | **Inclusion:** Children and young people (aged 18 years and younger) with first episode psychosis. Consideration will also be given to the specific needs of children and young people with psychosis and schizophrenia who have a mild learning disability and those from black and minority ethnic groups.  **Exclusion:** Study samples consisting only of individuals with a formal diagnosis of bipolar disorder. |
| *Intervention(s)* | - Cognitive behavioural therapy (CBT) - Counselling and supportive psychotherapy - Family intervention (including family therapy) - Psychodynamic psychotherapy and psychoanalysis - Psychoeducation - Social skills training - Arts therapies |
| *Comparison* | Alternative management strategies:   - Treatment as usual - Waitlist   Any of the above interventions offered as an alternative management strategy. |
| *Primary outcomes* | - Mental state (symptoms, depression, anxiety, mania) - Mortality (including suicide) - Global state - Psychosocial functioning - Social functioning - Leaving the study early for any reason - Remission |
| *Secondary outcomes* | None |
| *Electronic databases* | Core databases: Embase, MEDLINE, MEDLINE In-Process, PsycINFO  Topic specific databases and grey literature (see Appendix 8) |
| *Date searched* | Systematic reviews: 1995 to May 2012  RCT: inception of databases to May 2012 |
| *Study design* | RCTs; systematic reviews |
| *Review strategy* | - Two independent reviewers will review the full texts obtained through sifting all initial hits for their eligibility according to the inclusion criteria outlined in this protocol. - The initial approach is to conduct a meta-analysis evaluating the benefits and harms of psychological and psychosocial interventions. However, in the absence of adequate data, the literature will be presented via a narrative synthesis of the available evidence. - The main review will focus on children and young people between the ages of 14 and 18. The review will seek to identify whether modifications in treatment and management of children aged at or under 13 years and younger need to be made. Data from studies in which the study sample consists of children and young people under and over 18 years, but with a mean age of under 25, will be extrapolated if only limited evidence for children and young people aged 18 and younger is available. - Unpublished data will be included when the evidence is accompanied by a trial report containing sufficient detail to properly assess the quality of the data. The evidence must be submitted with the understanding that data from the study and a summary of the study’s characteristics will be published in the full guideline. Unpublished data will not be included when evidence submitted is commercial in confidence. |

Appendix B: Search strategy

*Medline search terms*

| 1 | delusions/ or hallucinations/ or exp "schizophrenia and disorders with psychotic features"/ or schizophrenia, childhood/ |
| --- | --- |
| 2 | (delusion$ or hallucinat$ or hebephreni$ or oligophreni$ or paranoi$ or psychotic$ or psychosis or psychoses or schizo$).ti,ab. |
| 3 | or/1-2 |
| 4 | adolescent/ or adolescent development/ or exp child/ or exp child development/ or exp infant/ or minors/ or exp puberty/ or puberty, delayed/ or puberty, precocious/ or expschools/ or students/ |
| 5 | (adolescen$ or child$ or infan$ or juvenile$ or teen$).hw. |
| 6 | (adolescen$ or baby or babies or boy$1 or child$ or delinquen$ or girl$1 or graders or infant$ or junior$1 or juvenile$ or kid$1 or kindergarten or minors or neonate$ or newborn$ or new born$ or p?ediatric$ or postpubert$ or postpubescen$ or prepubert$ or prepubescen$ or preschool$ or preteen$ or pubertal or puberty or puberties or pubescen$ or school$ or student$ or teen$ or toddler$ or (young$ adj2 (inpatient$ or patient$ or people$ or person$ or population$)) or youngster$ or youth$1).tw. |
| 7 | or/4-6 |
| 8 | 3 and 7 |

*Search Strategy For All Databases*

***1 Population search terms – all databases***

1.1 STEM – Mainstream Medical Databases

Embase, Medline, PreMEDLINE, PsycINFO – OVID SP

| 1 | exp psychosis/ or thought disorder/ |
| --- | --- |
| 2 | 1 use emez |
| 3 | delusions/ or hallucinations/ or exp "schizophrenia and disorders with psychotic features"/ or schizophrenia, childhood/ |
| 4 | 3 use mesz, prem |
| 5 | auditory hallucinations/ or delusions/ or hallucinations/ or hypnagogic hallucinations/ or paranoia/ or exp psychosis/ or schizoaffective disorder/ or thought disturbances/ or visual hallucinations/ |
| 6 | 5 use psyh |
| 7 | (delusion$ or hallucinat$ or hebephreni$ or oligophreni$ or paranoi$ or psychotic$ or psychosis or psychoses or schizo$).ti,ab. |
| 8 | or/2,4,6-7 |
| 9 | exp adolescence/ or exp adolescent/ or adolescent development/ or exp child/ or child development/ or exp childhood/ or disabled student/ or elementary student/ or high school student/ or high school/ or kindergarten/ or middle school student/ or middle school/ or expnewborn/ or nursery school/ or primary school/ or exp puberty/ or exp puberty disorders/ or school/ or school child/ or student/ |
| 10 | 9 use emez |
| 11 | adolescent/ or adolescent development/ or exp child/ or exp child development/ or exp infant/ or minors/ or exp puberty/ or puberty, delayed/ or puberty, precocious/ or expschools/ or students/ |
| 12 | 11 use mesz, prem |
| 13 | limit 8 to ((childhood or adolescence <13 to 17 years>) and (100 childhood or 120 neonatal or 140 infancy or 160 preschool age or 180 school age or 200 adolescence)) |
| 14 | adolescent attitudes/ or adolescent development/ or adolescent psychiatry/ or adolescent psychology/ or adolescent psychotherapy/ or adolescent psychopathology/ or boarding schools/ or charter schools/ or exp child development/ or child psychotherapy/ or child psychiatry/ or classmates/ or elementary schools/ or exp elementary school students/ or graduate schools/ or high school students/ or high schools/ or institutional schools/ or junior high school students/ or junior high schools/ or kindergarten students/ or kindergartens/ or middle schools/ or nongraded schools/ or nursery schools/ or exp preschool students/ or puberty/ or schools/ or special education students/ or students/ or vocational school students/ |
| 15 | 13 use psyh |
| 16 | 14 use psyh |
| 17 | or/15-16 |
| 18 | (adolescen$ or child$ or infan$ or juvenile$ or teen$).hw. |
| 19 | (adolescen$ or baby or babies or boy$1 or child$ or delinquen$ or girl$1 or graders or infant$ or junior$1 or juvenile$ or kid$1 or kindergarten or minors or neonate$ or newborn$ or new born$ or p?ediatric$ or postpubert$ or postpubescen$ or prepubert$ or prepubescen$ or preschool$ or preteen$ or pubertal or puberty or puberties or pubescen$ or school$ or student$ or teen$ or toddler$ or (young$ adj2 (inpatient$ or patient$ or people$ or person$ or population$)) or youngster$ or youth$1).tw. |
| 20 | or/10,12,17-19 |
| 21 | 8 and 20 |

1.2 STEM - topic specific databases

Allied and Complementary Medicine (AMED) – OVID SP

| 1 | delusions/ or hallucinations/ or psychotic disorders/ or schizophrenia/ |
| --- | --- |
| 2 | (delusion$ or hallucinat$ or hebephreni$ or oligophreni$ or paranoi$ or psychotic$ or psychosis or psychoses or schizo$).ti,ab. |
| 3 | 1 or 2 |
| 4 | adolescent/ or exp child/ or child development/ or education, special/ or exp infant/ or puberty/ or schools/ or students/ |
| 5 | (adolescen$ or child$ or infan$ or juvenile$ or teen$).hw. |
| 6 | (adolescen$ or baby or babies or boy$1 or child$ or delinquen$ or girl$1 or graders or infant$ or junior$1 or juvenile$ or kid$ or kindergarten or minors or neonate$ or newborn$ or new born$ or p?ediatric$ or postpubert$ or postpubescen$ or prepubert$ or prepubescen$ or preschool$ or preteen$ or pubertal or puberty or puberties or pubescen$ or school$ or student$ or teen$ or toddler$ or (young$ adj2 (inpatient$ or patient$ or people$ or person$ or population$)) or youngster$ or youth$1).tw. |
| 7 | or/4-6 |
| 8 | 3 and 7 |

1.3 STEM - topic specific databases

Australian Education Index (AEI), British Education Index (BEI), Education Resources in Curriculum (ERIC), Social Services Abstracts (SSA), Sociological Abstracts, Applied Social Sciences Index and Abstracts (ASSIA), International Bibliography of Social Sciences (IBSS) - ProQUEST

| s1 | all (delusion* or hallucinat* or hebephreni* or oligophreni* or paranoi* or psychotic* or psychosis or psychoses or schizo*) |
| --- | --- |
| s1 | all (delusion* or hallucinat* or hebephreni* or oligophreni* or paranoi* or psychotic* or psychosis or psychoses or schizo*) |
| s2 | all (adolescen* or baby or babies or boy or boyhood or boys or child* or delinquen* or girl or girls or girlhood or graders or infant* or junior or juniors or juvenile* or kid or kids or kindergarten or minors* or neonate* or newborn* or “new born*” or paediatric* or pediatric* or postpubert* or postpubescen* or prepubert* or prepubescen* or preschool* or preteen* or pubertal or puberty or puberties or pubescen* or school* or teen or teens or teenage* or toddler* or (young* near/2 (inpatient* or patient* or people* or person* or population*)) or youngster* or youth*) |
| s3 | s1 and s2 |

1.4 STEM - topic specific databases

CINAHL – EBSCO HOST

| s19 | s7 and s18 |
| --- | --- |
| s18 | s8 or s9 or s10 or s11 or s12 or s13 or s14 or s15 or s16 or s17 |
| s17 | ti ( (adolescen* or baby or babies or boy* or child* or delinquen* or girl* or graders or infant* or junior* or juvenile* or kid or kids or kindergarten or minors or neonate* or newborn* or “new born*” or paediatric* or pediatric* or postpubert* or postpubescen* or prepubert* or prepubescen* or preschool* or preteen* or pubertal or puberty or puberties or pubescen* or school* or student* or teen* or toddler* or (young* n2 (inpatient* or patient* or people* or person* or population*)) or youngster* or youth*) ) or ab ( (adolescen* or baby or babies or boy* or child* or delinquen* or girl* or graders or infant* or junior* or juvenile* or kid or kids or kindergarten or minors or neonate* or newborn* or “new born*” or paediatric* or pediatric* or postpubert* or postpubescen* or prepubert* or prepubescen* or preschool* or preteen* or pubertal or puberty or puberties or pubescen* or school* or student* or teen* or toddler* or (young* n2 (inpatient* or patient* or people* or person* or population*)) or youngster* or youth*) ) |
| s16 | mj (adolescen* or child* or infan* or juvenile* or teen*) |
| s15 | (mh "schools") or (mh "schools, special") or (mh "schools, secondary") or (mh "schools, nursery") or (mh "schools, middle") or (mh "schools, elementary") |
| s14 | (mh "students, disabled") |
| s13 | (mh "child development: adolescence (12-17 years) (iowanoc)") or (mh "child development: middle childhood (6-11 years) (iowanoc)") or (mh "child development: 5 years (iowanoc)") or (mh "child development: 4 years (iowanoc)") or (mh "child development: 3 years (iowanoc)") or (mh "child development: 2 years (iowanoc)") |
| s12 | (mh "students") or (mh "students, high school") or (mh "students, middle school") |
| s11 | (mh "puberty, delayed") or (mh "puberty, precocious") |
| s10 | (mh "puberty") |
| s9 | (mh "adolescent development") or (mh "child development") or (mh "infant development") |
| s8 | (mh "adolescence+") or (mh "child+") or (mh "minors (legal)") |
| s7 | s1 or s2 or s3 or s4 or s5 or s6 |
| s6 | ti ( (delusion* or hallucinat* or hebephreni* or oligophreni* or paranoi* or psychotic* or psychosis or psychoses or schizo*) ) or ab ( (delusion* or hallucinat* or hebephreni* or oligophreni* or paranoi* or psychotic* or psychosis or psychoses or schizo*) ) |
| s5 | (mh "psychotic disorders") |
| s4 | (mh "paranoid disorders") |
| s3 | (mh "schizoaffective disorder") or (mh "schizophrenia+") |
| s2 | (mh "hallucinations") or (mh "hallucination management (iowanic)") |
| s1 | (mh "delusions+") |

1.5 STEM - topic specific databases

HTA, CDSR, DARE, CENTRAL – Wiley

| #1 | mesh descriptor delusions, this term only |
| --- | --- |
| #2 | mesh descriptor hallucinations, this term only |
| #3 | mesh descriptor schizophrenia and disorders with psychotic features explode all trees |
| #4 | mesh descriptor schizophrenia, childhood, this term only |
| #5 | (delusion* or hallucinat* or hebephreni* or oligophreni* or paranoi* or psychotic* or psychosis or psychoses or schizo*):ti or (delusion* or hallucinat* or hebephreni* or oligophreni* or paranoi* or psychotic* or psychosis or psychoses or schizo*):ab |
| #6 | ([2011 or #2 or #3 or #4 or #5](#_ENREF_53))([2011 or #2 or #3 or #4 or #5](#_ENREF_52))([2011 or #2 or #3 or #4 or #5](#_ENREF_52))([2011 or #2 or #3 or #4 or #5](#_ENREF_52))([2011 or #2 or #3 or #4 or #5](#_ENREF_52))([2011 or #2 or #3 or #4 or #5](#_ENREF_52))([2011 or #2 or #3 or #4 or #5](#_ENREF_52))([2011 or #2 or #3 or #4 or #5](#_ENREF_52))([2011 or #2 or #3 or #4 or #5](#_ENREF_52))([2011 or #2 or #3 or #4 or #5](#_ENREF_52))([2011 or #2 or #3 or #4 or #5](#_ENREF_51))([2011 or #2 or #3 or #4 or #5](#_ENREF_51))([2011 or #2 or #3 or #4 or #5](#_ENREF_51))([2011 or #2 or #3 or #4 or #5](#_ENREF_52))([2011 or #2 or #3 or #4 or #5](#_ENREF_51))([2011 or #2 or #3 or #4 or #5](#_ENREF_51))([2011 or #2 or #3 or #4 or #5](#_ENREF_51))([2011 or #2 or #3 or #4 or #5](#_ENREF_51))([2011 or #2 or #3 or #4 or #5](#_ENREF_51)) |
| #7 | mesh descriptor adolescent, this term only |
| #8 | mesh descriptor child explode all trees |
| #9 | mesh descriptor infant explode all trees |
| #10 | mesh descriptor adolescent development, this term only |
| #11 | mesh descriptor child development explode all trees |
| #12 | mesh descriptor minors, this term only |
| #13 | mesh descriptor puberty, delayed, this term only |
| #14 | mesh descriptor puberty, precocious, this term only |
| #15 | mesh descriptor students, this term only |
| #16 | mesh descriptor schools, this term only |
| #17 | mesh descriptor puberty, this term only all trees |
| #18 | (adolescen* or child* or infan* or juvenile* or teen*):kw or (adolescen* or baby or babies or boy* or child* or delinquen* or girl* or graders or infant* or junior* or juvenile* or kid or kids or kindergarten or minors or neonate* or newborn* or new born* or pediatric* or paediatric* or postpubert* or postpubescen* or prepubert* or prepubescen* or preschool* or preteen* or pubertal or puberty or puberties or pubescen* or school* or student* or teen* or toddler* or (young* near/2 (inpatient* or patient* or people or person* or population)) or youngster* or youth*):ti or (adolescen* or baby or babies or boy* or child* or delinquen* or girl* or graders or infant* or junior* or juvenile* or kid or kids or kindergarten or minors or neonate* or newborn* or new born* or pediatric* or paediatric* or postpubert* or postpubescen* or prepubert* or prepubescen* or preschool* or preteen* or pubertal or puberty or puberties or pubescen* or school* or student* or teen* or toddler* or (young* near/2 (inpatient* or patient* or people or person* or population)) or youngster* or youth*):ab |
| #19 |  |
| #20 |  |

1.6 STEM - topic specific databases

SSCI – Web of Knowledge

| #1 | (topic=(delusion* or hallucinat* or hebephreni* or oligophreni* or paranoi* or psychotic* or psychosis or psychoses or schizo*)) or (title=(delusion* or hallucinat* or hebephreni* or oligophreni* or paranoi* or psychotic* or psychosis or psychoses or schizo*)) |
| --- | --- |
| #2 | (topic=(adolescen* or baby or babies or boy or boyhood or boys or child* or delinquen* or girl or girls or girlhood or graders or infant* or junior or juniors or juvenile* or kindergarten or minors or neonate* or newborn* or “new born*” or paediatric* or pediatric* or postpubert* or postpubescen* or prepubert* or prepubescen* or preschool* or preteen* or pubertal or puberty or puberties or pubescen* or school* or teen or teens or teenage* or toddler* or (young* near (inpatient* or patient* or people or person* or population)) or youngster* or youth*)) or (title=(adolescen* or baby or babies or boy or boyhood or boys or child* or delinquen* or girl or girls or girlhood or graders or infant* or junior or juniors or juvenile* or kid or kids or kindergarten or minors or neonate* or newborn* or “new born*” or paediatric* or pediatric* or postpubert* or postpubescen* or prepubert* or prepubescen* or preschool* or preteen* or pubertal or puberty or puberties or pubescen* or school* or teen or teens or teenage* or toddler* or (young* near (inpatient* or patient* or people or person* or population)) or youngster* or youth*)) |
| #3 | (topic=(“young* inpatient*” or “young* patient” or “young* people” or “young* population*”)) or (title=(“young* inpatient*” or “young* patient” or “young* people” or “young* population*”)) |
| #4 | #2 or #3 |
| #5 | #1 and #4 |

1.7STEM - grey literature databases

Health Management Information Consortium (HMIC), PsycBOOKS, PsycEXTRA – OVID SP [high spec]

| 1 | ((delusion$ or hallucinat$ or hebephreni$ or oligophreni$ or paranoi$ or psychotic$ or psychosis or psychoses or schizo$) and (adolescen$ or baby or babies or boy$1 or child$ or delinquen$ or girl$1 or graders or infant$ or junior$1 or juvenile$ or kid or kids or kindergarten or minors or neonate$ or newborn$ or new born$ or paediatric* or pediatric* or postpubert$ or postpubescen$ or prepubert$ or prepubescen$ or preschool$ or preteen$ or pubertal or puberty or puberties or pubescen$ or school$ or student$ or teen$ or toddler$ or (young$ adj2 (inpatient$ or patient$ or people$ or person$ or population$)) or youngster$ or youth$1)).ti,ab,hw. |
| --- | --- |

***2 Study design filters – all databases***

**2.1 Systematic review study design filters**

2.1.1 Systematic review study design filter

Embase, Medline, PreMEDLINE, PsycINFO – OVID SP

| 1 | meta analysis/ or systematic review/ |
| --- | --- |
| 2 | 1 use emez |
| 3 | meta analysis.sh,pt. or "meta-analysis as topic"/ or "review literature as topic"/ |
| 4 | 3 use mesz, prem |
| 5 | (literature review or meta analysis).sh,id,md. or systematic review.id,md. |
| 6 | 5 use psyh |
| 7 | (exp bibliographic database/ or (((electronic or computer$ or online) adj database$) or bids or cochrane or embase or index medicus or isi citation or medline or psyclit or psychlit or scisearch or science citation or (web adj2 science)).ti,ab.) and (review$.ti,ab,sh,pt. or systematic$.ti,ab.) |
| 8 | 7 use emez |
| 9 | (exp databases, bibliographic/ or (((electronic or computer$ or online) adj database$) or bids or cochrane or embase or index medicus or isi citation or medline or psyclit or psychlit or scisearch or science citation or (web adj2 science)).ti,ab.) and (review$.ti,ab,sh,pt. or systematic$.ti,ab.) |
| 10 | 9 use mesz, prem |
| 11 | (computer searching.sh,id. or (((electronic or computer$ or online) adj database$) or bids or cochrane or embase or index medicus or isi citation or medline or psyclit or psychlit or scisearch or science citation or (web adj2 science)).ti,ab.) and (review$.ti,ab,pt. or systematic$.ti,ab.) |
| 12 | 11 use psyh |
| 13 | ((analy$ or assessment$ or evidence$ or methodol$ or quantitativ$ or systematic$) adj2 (overview$ or review$)).tw. or ((analy$ or assessment$ or evidence$ or methodol$ or quantitativ$ or systematic$).ti. and review$.ti,pt.) or (systematic$ adj2 search$).ti,ab. |
| 14 | (metaanal$ or meta anal$).ti,ab. |
| 15 | (research adj (review$ or integration)).ti,ab. |
| 16 | reference list$.ab. |
| 17 | bibliograph$.ab. |
| 18 | published studies.ab. |
| 19 | relevant journals.ab. |
| 20 | selection criteria.ab. |
| 21 | (data adj (extraction or synthesis)).ab. |
| 22 | (handsearch$ or ((hand or manual) adj search$)).ti,ab. |
| 23 | (mantel haenszel or peto or dersimonian or der simonian).ti,ab. |
| 24 | (fixed effect$ or random effect$).ti,ab. |
| 25 | ((pool$ or combined or combining) adj2 (data or trials or studies or results)).ti,ab. |
| 26 | or/2,4,6,8,10,12-25 |

2.1.2 Systematic review study design filter
AMED – OVID SP

| 1 | meta analysis/ |
| --- | --- |
| 2 | (databases bibliographic/ or (((electronic or computer$ or online) adj database$) or bids or cochrane or embase or index medicus or isi citation or medline or psyclit or psychlit or scisearch or science citation or (web adj2 science)).ti,ab.) and (review$.ti,ab,pt. or systematic$.ti,ab.) |
| 3 | ((analy$ or assessment$ or evidence$ or methodol$ or qualitativ$ or quantativ$ or systematic$) adj2 (overview$ or review$)).tw. or ((analy$ or assessment$ or evidence$ or methodol$ or quantativ$ or qualitativ$ or systematic$).ti. and review$.ti,pt.) or (systematic$ adj2 search$).ti,ab. |
| 4 | (metaanal$ or meta anal$).ti,ab. |
| 5 | (research adj (review$ or integration)).ti,ab. |
| 6 | reference list$.ab. |
| 7 | published studies.ab. |
| 8 | relevant journals.ab. |
| 9 | selection criteria.ab. |
| 10 | (data adj (extraction or synthesis)).ab. |
| 11 | (handsearch$ or ((hand or manual) adj search$)).ti,ab. |
| 12 | (mantel haenszel or peto or dersimonian or der simonian).ti,ab. |
| 13 | (fixed effect$ or random effect$).ti,ab. |
| 14 | or/1-13 |

2.1.3 Systematic review study design filter

Australian Education Index (AEI), British Education Index (BEI), Education Resources in Curriculum (ERIC), Social Services Abstracts (SSA), Sociological Abstracts, Applied Social Sciences Index and Abstracts (ASSIA), International Bibliography of Social Sciences (IBSS) - ProQUEST

| **S1** | all ((“meta anal*” or “systematic overview” or “systematic review” or “systematic search”)) |
| --- | --- |

2.1.4 Systematic review study design filter

CINAHL – EBSCO HOST

| # | query |
| --- | --- |
| s33 | s1 or s2 or s3 or s4 or s5 or s6 or s7 or s8 or s9 or s10 or s11 or s12 or s13 or s14 or s15 or s16 or s22 or s23 or s26 or s27 or s28 or s29 or s30 or s31 or s32 |
| s32 | ti ( analy* n5 review* or assessment* n5 review* or evidence* n5 review* or methodol* n5 review* or quantativ* n5 review* or systematic* n5 review* ) or ab ( analy* n5 review* or assessment* n5 review* or evidence* n5 review* or methodol* n5 review* or quantativ* n5 review* or systematic* n5 review* ) |
| s31 | ti ( analy* n5 overview* or assessment* n5 overview* or evidence* n5 overview* or methodol* n5 overview* or quantativ* n5 overview* or systematic* n5 overview* ) or ab ( analy* n5 overview* or assessment* n5 overview* or evidence* n5 overview* or methodol* n5 overview* or quantativ* n5 overview* or systematic* n5 overview* ) |
| s30 | ti ( pool* n2 results or combined n2 results or combining n2 results ) or ab ( pool* n2 results or combined n2 results or combining n2 results ) |
| s29 | ti ( pool* n2 studies or combined n2 studies or combining n2 studies ) or ab ( pool* n2 studies or combined n2 studies or combining n2 studies ) |
| s28 | ti ( pool* n2 trials or combined n2 trials or combining n2 trials ) or ab ( pool* n2 trials or combined n2 trials or combining n2 trials ) |
| s27 | ti ( pool* n2 data or combined n2 data or combining n2 data ) or ab ( pool* n2 data or combined n2 data or combining n2 data ) |
| s26 | s24 and s25 |
| s25 | ti review* or pt review* |
| s24 | tianaly* or assessment* or evidence* or methodol* or quantativ* or systematic* |
| s23 | ti “systematic* n5 search*” or ab “systematic* n5 search*” |
| s22 | (s17 or s18 or s19) and (s20 or s21) |
| s21 | ti systematic* or ab systematic* |
| s20 | tx review* or mw review* or pt review* |
| s19 | (mh "cochrane library") |
| s18 | ti ( bids or cochrane or index medicus or “isi citation” or psyclit or psychlit or scisearch or “science citation” or web n2 science ) or ab ( bids or cochrane or index medicus or “isi citation” or psyclit or psychlit or scisearch or “science citation” or web n2 science ) |
| s17 | ti ( “electronic database*” or “bibliographic database*” or “computeri?ed database*” or “online database*” ) or ab ( “electronic database*” or “bibliographic database*” or “computeri?ed database*” or “online database*” ) |
| s16 | (mh "literature review") |
| s15 | pt systematic* or pt meta* |
| s14 | ti ( “fixed effect*” or “random effect*” ) or ab ( “fixed effect*” or “random effect*” ) |
| s13 | ti ( “mantel haenszel” or peto or dersimonian or “der simonian” ) or ab ( “mantel haenszel” or peto or dersimonian or “der simonian” ) |
| s12 | ti ( handsearch* or "hand search*" or "manual search*" ) or ab ( handsearch* or "hand search*" or "manual search*" ) |
| s11 | ab "data extraction" or "data synthesis" |
| s10 | ab "selection criteria" |
| s9 | ab "relevant journals" |
| s8 | ab "published studies" |
| s7 | abbibliograph* |
| s6 | ab "reference list*" |
| s5 | ti ( “research review*” or “research integration” ) or ab ( “research review*” or “research integration” ) |
| s4 | ti ( metaanal* or “meta anal*”) or ab ( metaanal* or “meta anal*”) |
| s3 | (mh "meta analysis") |
| s2 | (mh "systematic review") |
| s1 | (mh "literature searching+") |

2.1.5 S ystematic review study design filter

SSCI – Web of Knowledge

| #1 | title=(“electronic database*” or “computer* database*” or “online database*” or bids or cochrane or embase or “index medicus” or “isi citation” or medline or psyclit or psychlit or scisearch or “science citation” or “web of science”) |
| --- | --- |
| #2 | title=(review* or systematic*) or topic=(review* or systematic*) |
| #3 | #1 and #2 |
| #4 | topic=((systematic* near search* or metaanal* or “meta anal*” or “research review*” or “research integration” or “reference list*” or bibliograph* or “published studies” or “relevant journals” or “selection criteria” or “data extraction” or “data synthesis” or handsearch* or “hand search*” or “manual search*” or “mantel haenszel” or peto or dersimonian or “der simonian” or “fixed effect*” or “random effect*” or ((pool* or combined or combining) near (data or trials or studies or results)))) or title=((systematic* near search* or metaanal* or “meta anal*” or “research review*” or “research integration” or “reference list*” or bibliograph* or “published studies” or “relevant journals” or “selection criteria” or “data extraction” or “data synthesis” or handsearch* or “hand search*” or “manual search*” or “mantel haenszel” or peto or dersimonian or “der simonian” or “fixed effect*” or “random effect*”) or ((pool* or combined or combining) near (data or trials or studies or results)))) |
| #5 | topic=(((analy* or assessment* or evidence* or methodol* or quantitativ* or systematic*) near (overview* or review*))) or title=(((analy* or assessment* or evidence* or methodol* or qualitativ* or quantitativ* or systematic*) near (overview* or review*))) |
| #6 | #3 or #4 or #5 |

**2.2 Randomised controlled trial study design filters**

2.2.1 Randomised controlled trial study design filter

Embase, Medline, PreMEDLINE, PsycINFO – OVID SP

| 1 | exp "clinical trial (topic)"/ or exp clinical trial/ or crossover procedure/ or double blind procedure/ or placebo/ or randomization/ or random sample/ or single blind procedure/ |
| --- | --- |
| 2 | 1 use emez |
| 3 | exp clinical trial/ or cross-over studies/ or double-blind method/ or placebos/ or random allocation/ or "randomized controlled trials as topic"/ or single-blind method/ |
| 4 | 3 use mesz, prem |
| 5 | (clinical trials or placebo or random sampling).sh,id. |
| 6 | 5 use psyh |
| 7 | (clinical adj2 trial$).ti,ab. |
| 8 | (crossover or cross over).ti,ab. |
| 9 | (((single$ or doubl$ or trebl$ or tripl$) adj2 blind$) or mask$ or dummy or doubleblind$ or singleblind$ or trebleblind$ or tripleblind$).ti,ab. |
| 10 | (placebo$ or random$).ti,ab. |
| 11 | treatment outcome$.md. use psyh |
| 12 | animals/ not human$.mp. use emez |
| 13 | animal$/ not human$/ use mesz, prem |
| 14 | (animal not human).po. use psyh |
| 15 | (or/2,4,6-11) not (or/12-14) |

2.2.2 Randomised controlled trial study design filter

AMED – OVID SP

| 1 | (clinical trials or double blind method or placebos or random allocation).sh. |
| --- | --- |
| 2 | trial$.ti,ab. |
| 3 | (crossover or cross over).ti,ab. |
| 4 | (((single$ or doubl$ or trebl$ or tripl$) adj5 blind$) or mask$ or dummy or singleblind$ or doubleblind$ or trebleblind$ or tripleblind$).ti,ab. |
| 5 | (placebo$ or random$).ti,ab. |
| 6 | or/1-6 |

2.2.3 Randomised controlled trial study design filter

Australian Education Index (AEI), British Education Index (BEI), Education Resources in Curriculum (ERIC), Social Services Abstracts (SSA), Sociological Abstracts, Applied Social Sciences Index and Abstracts (ASSIA), International Bibliography of Social Sciences (IBSS) – PRO QUEST

| S1 | all ((clinical near/1 trial* or crossover or “cross over” ) or ((single* or doubl* or trebl* or tripl*) near/1 (blind* or mask* or dummy)) or (singleblind* or doubleblind* or trebleblind* or tripleblind* or placebo* or random*) ) |
| --- | --- |

2.2.4 Randomised controlled trial study design filter

SSCI – Web of Knowledge

| #1 | topic=(((clinical near trial* or crossover or “cross over”) or ((single* or doubl* or trebl* or tripl*) near (blind* or mask* or dummy)) or (singleblind* or doubleblind* or trebleblind* or tripleblind* or placebo* or random*))) or title=(((clinical near trial* or crossover or “cross over”) or ((single* or doubl* or trebl* or tripl*) near (blind* or mask* or dummy)) or (singleblind* or doubleblind* or trebleblind* or tripleblind* or placebo* or random*))) |
| --- | --- |

Appendix C: Excluded studies

| **Reference** | **Exclusion Reason** |
| --- | --- |
| Ananth JV, Vacaflor L, Kekhwa G, Sterlin C, Ban TA (1972) Nicotinic acid in the treatment of newly admitted schizophrenic patients: a placebo-controlled study. Int Z Klin Pharmakol Ther Toxikol 5: 406-410 | No relevant medication |
| Bao X Q (1988) A double-blind study on the effect of clozapine penfluridol and chlorpromazine in the treatment of schizophrenia. Chinese Journal of Neurology and Psychiatry 21: 274-276+318 | Adult population |
| Bildik T, Ozbaran NB, Kose S, Cetin SK (2012) Effectiveness and tolerability of aripiprazole in a real-world outpatient population of youth. Klinik Psikofarmakoloji Bülteni 22: 225-234 | Non RCT |
| Charalampous KD, Freemesser GF, Malev J, Ford K (1974) Loxapine succinate: a controlled double-blind study in schizophrenia. Curr Ther Res Clin Exp 16: 829-837 | Drug not licensed in the UK |
| Chaudry IB, Hallak J, Husain N, Minhas F, Stirling J, et al. (2012) Minocycline benefits negative symptoms in early schizophrenia: a randomised double-blind placebo-controlled clinical trial in patients on standard treatment. J Psychopharmacol 26: 1185-1193 | Adult population |
| Ciurezu T, Ionescu R, Nica Udangiu S, Niturad D, Oproiu L, et al. (1976) Double-blind clinical study of HF 1854 (LX 100-129, clozapine or leponex) as compared with haloperidol. Neurologie et Psychiatrie 14: 29-34 | Adult population |
| Claghorn JL (1972) A double-blind comparison of haloperidol (Haldol) and thioridazine (Mellaril) in outpatient children. Curr Ther Res Clin Exp 14: 785-789 | Drug discontinued in 2005 |
| De Sousa RT, Busnello JV Forlenza OV, Zanetti MV, Soeiro-de-Souza MG, et al. (2012) Early improvement of psychotic symptoms with lithium monotherapy as a predictor of later response in mania. J Psychiatr Res 46: 1564-1568 | Non RCT |
| Escande M, Granier F, Gardes JP, Boscredon J, Concina M (1983) Clinical trial of loxapine succinate in the treatment of 30 cases of psychotic states. Ann Med Psychol (Paris) 141: 309-322 | Drug not licensed in the UK |
| Feng-Ju Y, Fu-Gen S, Zhi-Hua Z (2006) Short-term curative effect of electroacupuncture as an adjunctive treatment on schizophrenia. Chinese Journal of Integrated Traditional and Western Medicine 26: 253-255 | Outside scope: Acupuncture |
| Freedman R, Kirch D, Bell J, Adler LE, Pecevich M, et al. (1982) Clonidine treatment of schizophrenia. Double-blind comparison to placebo and neuroleptic drugs. Acta Psychiatr Scand 65: 35-45 | Sample size |
| Freeman H, Oktem N, Oktem MR (1968) A double-blind study of SKF 14336 vs. trifluoperazine in schizophrenic patients. Curr Ther Res Clin Exp 10: 537-542 | Adult population |
| Geffen Y, Keefe R, (2012) Bl-1020, a new -aminobutyric acid-enhanced antipsychotic: results of 6-week, randomized, double-blind, controlled, efficacy and safety study. J Clin Psychiatry 73: e1168-1174 | Adult population |
| Gleeson JFM., Chanen A, Cotton SM, Pearce T, Newman B, et al. (2012) Treating co-occurring first-episode psychosis and borderline personality: A pilot randomized controlled trial. Early Interv Psychiatry 6: 21-29 | Borderline personality disorder with psychosis |
| Goulet JL (1993) Effect of neuroleptic Medication Management Module in young psychotics. Can J Psychiatry 38: 571-573 | No relevant medication |
| Huang MW, Yang TT, (2012). Effects of paliperidone extended release on the symptoms and functioning of schizophrenia. BMC Clinical Pharmacology 12: 1  doi: 10.1186/1472-6904-12-1 | Adult population |
| Jaugey L, Urben S, Pihet S, Halfon O, Holzer L (2012) Short-and long-term outcomes of a randomized controlled trial of a computer-assisted cognitive remediation (CACR) program in adolescents with psychosis or at high risk of psychosis. Biol Psychiatry 71: Suppl., 84, 1S-106S. 67th Annual Scientific Convention and Meeting | Population ‘at risk’ |
| Kane JM, Sanchez R, Perry PP, Jin N, Johnson BR, Forbes RA, et al. (2012). Aripiprazole intramuscular depot as maintenance treatment in patients with schizophrenia: a 52-week, multicenter, randomized, double-blind, placebo-controlled study. J Clin Psychiatry 73: 617-624 | Adult population |
| Leavey G, Gulamhussein S, Papadopoulos C, Johnson-Sabine E, et al. (2004) A randomized controlled trial of a brief intervention for families of patients with a first episode of psychosis. Psychol Med 34: 423-431 | Intervention (for carers only) |
| Nishizono M, Matsuki K (1984) Clinical effects of bromperidol on schizophrenia. Kyushu Neuropsychiatry 30, 373-382 | Adult population |
| Parent M, Toussaint C, Parent M (1982) Flupenthixol versus haloperidol in acute psychotic episodes. Acta Psychiatr Belg 82: 617-631 | Adult population |
| Proselkova ME, Uzhanov NV (1991) Experience with using etaperazine (perphenazine) in the treatment of patients with schizophrenia in a preschool psychiatric department. Zhurnal Nevropatologii i Psikhiatrii Imeni S. S. Korsakova 91, 120-122 | Non RCT |
| Puig O, Baeza I, Sanchez V (2009) Memory improvements after cognitive remediation therapy in adolescents with schizophrenia. Eur Arch Psy Clin N 259 (Suppl. 1): 88 | Sample size |
| Rondeau MC, Rho A, Iyer S, Joober R, Schmitz N, et al. (2012) A randomized controlled evaluation of 'extended specialized early intervention service' vs. 'regular care' for long-term management of early psychosis. Early Interv Psychiatry 6: 72 | Intervention: not pharmacological or psychological |
| Schmidt SJ, Roder V (2012). Psychosocial treatments in schizophrenia: Update and future directions. Minerva Psichiatr 53: 217-231 | Design: non-systematic review |
| Stain HJ, Crittenden K, Startup M (2010) The depth randomised controlled trial of cognitive behaviour therapy for youth at ultra high risk for psychosis: Baseline characteristics for rural and urban youth. Aust Nz J Psychiat 44 (Suppl. 1): 25-26 | Population ‘at risk’ |
| Stain HJ, Crittenden K, Startup M (2011) Rural and urban youth at ultra high risk for psychosis: Baseline characteristics from the depth randomised controlled trial of cognitive behavior therapy. Schizophr Bull 37 (Suppl. 1): 322 | Population ‘at risk’ |
| Takekita Y, Kato M, Wakeno M, Sakai S, Suwa A, et al. (2013) A 12-week randomized, open-label study of perospirone versus aripiprazole in the treatment of Japanese schizophrenia patients. Prog Neuropsychopharmacol Biol Psychiatry 40: 110-114. | Adult population |
| Zhang JP, Gallego JA, Robinson DG, Malhotra AK, Kane JM, et al. (2012) Efficacy and safety of individual second-generation vs. first-generation antipsychotics in first-episode psychosis: a systematic review and meta-analysis. Int J Neuropsychop doi:10.1017/S1461145712001277 | Relevant trials included |
| Zhao T, Park TW, Yang JC, Juang GB, Kim MG, et al. (2012) Efficacy and safety of ziprasidone in the treatment of first-episode psychosis: an 8-week, open-label, multicenter trial. Int J Neuropsychop 27: 184-190 | Adult population |

Appendix D: Studies awaiting assessment

| Reference | Reason |
| --- | --- |
| Gao C (2007) A comparative study of risperidone and perphenazine in the treatment of child schizophrenia. Chinese Journal of Health Psychology 15: 950-951 | Non-English language report |
| Huo WH, Ma YB, Li GZ (2007) A controlled study of risperidone in child schizophrenia. Medical Journal of Chinese People's Health 19: 472-473 | Non-English language report |
| Kaleda VG, Oleichik IV, Artioukh VV (2000) Risperidone vs haloperidol in the therapy of adolescent schizophrenia and schizoaffective disorders: an open comparative medium-term efficacy and tolerability study. Int J Neuropsychop 3, 99 | Conference abstract |
| Saija CC, Guastella AJ, Langdon R, Ward PB, Naismith SL, et al. (2012) The impact of social cognition training and oxytocin on social functioning in early psychosis. Early Interv Psychiatry 6: 82 | Conference abstract |
| Shahrivar ZJ, Alaghband-rad, Gharaie JM, Seddigh A, Salesian N, et al. (2012) The efficacy of an integrated treatment in comparison with treatment as usual in a group of children and adolescents with first-episode psychosis during a two -year follow-up. Iranian Journal of Psychiatry and Clinical Psychology 18: 124-127 | Non-English language report |
| Srihari V, Phutane V, Breitborde N, Tek C, Woods S (2012) Early intervention for psychosis in the U.S. public sector: A pragmatic randomized controlled trial. Early Interv Psychiatry 6: 106 | Conference abstract |
| Wiesel FA, Alfredsson G, Bjerkenstedt L (1985) Dogmatil for the treatment of negative symptoms in schizophrenic patients. Semaine des Hopitaux 61: 1317-1321 | Non-English language report |
| Zhang CJ, Li C, Yang FS, Yang RL (2005) Effect of health education for parents in preventing the recurrence of schizophrenia in their children after first episode. Chinese Journal of Clinical Rehabilitation 9: 19-21 | Non-English language report |
| Xie Q, Feng SH (2012). Efficacies of paliperidone extended-release tablets and olanzapine for treating schizophrenia in children and adolescents. Chinese Journal of New Drugs 21: 1916-1919 | Non-English language report |

Appendix E: On-going studies

| **Reference** |
| --- |
| Morrison AP, Wardle, Hutton P, Davies L, Dunn G, et al. (2013) Assessing cognitive therapy instead of neuroleptics: Rationale, study design and sample characteristics of the ACTION trial. Psychosis: Psychological, Social and Integrative Approaches 5: 82-92 |
| Datta SS, Kumar A, Wright SD, Russell P (2012) Typical antipsychotics for psychosis in adolescents (Protocol). Cochrane Database of Systematic Reviews 2013, The Cochrane Collaboration. |
| Kumar A, Datta SS, Wright SD, Furtado VA, Russell P (2012) Atypical antipsychotics for psychosis in adolescents (Protocol). Cochrane Database of Systematic Reviews 2013, The Cochrane Collaboration. |
| Wright SD, Datta SS, Kumar A (2012) Psychological interventions for psychosis in adolescents (Protocol). Cochrane Database of Systematic Reviews 2013, The Cochrane Collaboration. |

Appendix F: Description of included psychological and psychosocial interventions

| **Study ID** | **Intervention** | **Frequency/Duration** | **Description** |
| --- | --- | --- | --- |
| APTER1978* | Movement therapy | 3 sessions a week lasting for one hour each, over 12 weeks. | Movement therapy. “The idea of movement therapy is that the patient becomes 'aware' of his body, is able to know the full limits of his physical potential and is able to realize their potential completely. There are six main foci of attention: body stability, improving body image, coordination, expression of body energy, organisation and planning of body movements and non-verbal communication and expression.”[1](p.156) |
| JACKSON2008 | Individual CBT | Maximum 20 sessions over 14 weeks. | Active Cognitive Therapy for Early Psychosis (ACE). “ACE therapy involved an assessment of the presenting psychotic and non-psychotic complaints followed by a formulation of the relationship between these complaints and the participant’s life history. Each area of difficulty was treated from a broadly cognitive behavioural perspective.”[2](p.727-728) |
| JACKSON2009 | Individual CBT | Maximum 26 sessions over 26 weeks. | Cognitive therapy based recovery intervention (CRI). “There were three key components: (a) engagement and formulation; (b) trauma processing; and (c) appraisals of psychotic illness (shame, loss and entrapment).”[3](p.456) |
| HADDOCK2006* | Individual CBT | 15–20 hours within a 5-week period, plus ‘booster’ sessions at a further 2 weeks and 1, 2 and 3 months. | CBT. “The cognitive-behavioural treatment followed a detailed treatment manual designed by the authors and used in earlier CBT for psychosis research studies. It was primarily focused on the treatment of auditory hallucinations and delusions, associated symptoms and problems (for example, anxiety, depression, self-esteem) and relapse prevention.”[4](p.255) |
| MAK2007* | Individual CBT | Fortnightly sessions lasting for one hour each, over a period of 65 weeks. | CBT. “Psychological treatment was based on clinical assessments of the subjects’ presenting problems and needs; the orientation being cognitive-behavioural.”[5](p.18) |
| POWER2003 | Individual CBT | 8-10 sessions over a period of 10 weeks. | LifeSPAN therapy. “It draws on the experience at EPPIC with Cognitive Oriented Therapy for Early Psychosis (COPE) and suicide prevention manuals such as *Choosing to live* and *Cognitive therapy of suicide behaviour: a manual for treatment*. There were four phases: initial engagement; suicide risk assessment/formulation; cognitive modules; and final closure/handover.”[6](p.417) |
| GLEESON2009 | Family CBT + individual CBT | Fortnightly sessions over a period of 7 months. | Combined individual and family CBT for relapse prevention (RPT). “Key differences between TAU and RPT included (1) the shared, written individualized formulation regarding relapse risk; (2) the systematic and phased approach to relapse prevention via a range of cognitive behavioral interventions; (3) the parallel individual and family sessions focused on relapse prevention; and (4) supervision specifically focused on relapse prevention.”[7](p.479) |
| LINSZEN1996 | Family CBT | 18 sessions over a period of 12 months. | In-patient psychosocial and behavioural family intervention (IPFI). “A behavioural family intervention was added to the in-patient psycho-social intervention. The family treatment was based on the behavioural family management approach as developed by Falloon *et al*. (1984). Psychoeducation, communication training and the development of problem solving skills were the main components; the methods include instruction, role rehearsal and modelling.”[8](p.336-337) |
| *Note.*  ** Data not presented in a form that could be used in a meta-analysis*  CBT = Cognitive Behavioural Therapy; EPPIC = Early Psychosis Prevention and Intervention Centre; TAU = Treatment As Usual  1. Apter A, Sharir I, Tyano S, Wijsenbeek H (1978) Movement therapy with psychotic adolescents. Br J Med Psychol 51: 155-159.  2. Jackson HJ, McGorry PD, Killackey E, Bendall S, Allott K, et al. (2008) Acute-phase and 1-year follow-up results of a randomized controlled trial of CBT versus Befriending for first-episode psychosis: the ACE project. Psychol Med 38: 725-735.  3. Jackson C, Trower P, Reid I, Smith J, Hall M, et al. (2009) Improving psychological adjustment following a first episode of psychosis: a randomised controlled trial of cognitive therapy to reduce post psychotic trauma symptoms. Behav Res Ther 47: 454-462.  4. Haddock G, Lewis S, Bentall R, Dunn G, Drake R, et al. (2006) Influence of age on outcome of psychological treatments in first-episode psychosis. Br J Psychiatry 188: 250-254.  5. Mak GKL, Li FWS, Lee PWH (2007) A pilot study on psychological interventions with Chinese young adults with schizophrenia Hong Kong Journal of Psychiatry 17: 17-23.  6. Power PJ, Bell RJ, Mills R, Herrman-Doig T, Davern M, et al. (2003) Suicide prevention in first episode psychosis: the development of a randomised controlled trial of cognitive therapy for acutely suicidal patients with early psychosis. Aust N Z J Psychiatry 37: 414-420.  7. Gleeson JF, Cotton SM, Alvarez-Jiménez M, Wade D, Gee D, et al. (2009) A randomized controlled trial of relapse prevention therapy for first-episode psychosis patients. J Clin Psychiatry 70: 477-486.  8. Linszen D, Dingemans P, Van der Does JW, Nugter A, Scholte P, et al. (1996) Treatment, expressed emotion and relapse in recent onset schizophrenic disorders. Psychol Med 26: 333-342. | | | |

Appendix G: Risk of bias assessments for included trials

*Pharmacological interventions trials*

*
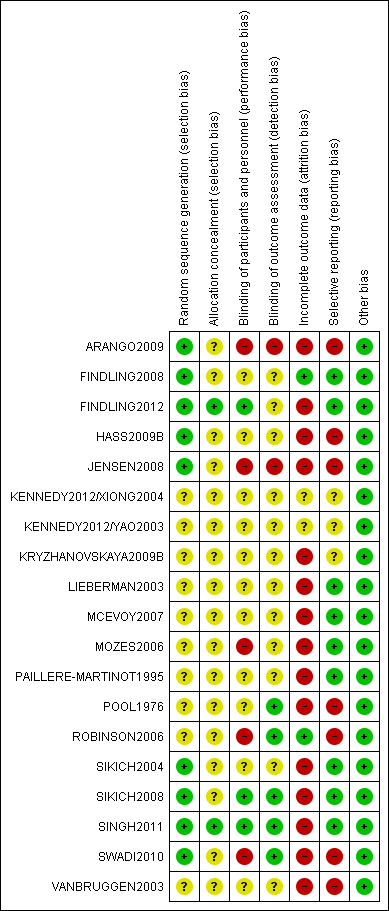
*

*Psychological interventions trials*

*
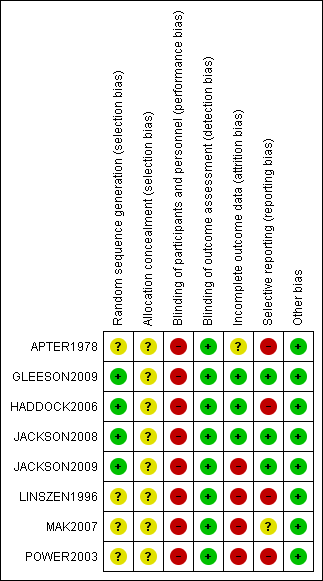
*

Appendix H: Summary of effects

**Pharmacological Interventions**

Antipsychotic medication versus placebo at post treatment

| **Outcome** | **Trials in analysis** | **Participants in analysis** | **Effect Estimate [95% CI],Random-effects** | **Heterogeneity:I^2^; Chi² (p value)** | **Quality** |
| --- | --- | --- | --- | --- | --- |
| **Total symptoms** | **4 (57%)** | **782 (77%)** | **SMD=-0.42 [-0.58, -0.26]** | **0%; 4.00,(P = 0.68)** | **Low^3,4^** |
| *Sensitivity analysis** | *3 (43%)* | *675 (66%)* | *SMD= -0.39 [-0.56, -0.22]* | *0%, 3.14, (P=0.94)* | *-* |
| *Subgroup analysis:*  *‘Lower dose’* | 4 (57%) | 444 (74%) | SMD=-0.34 [-0.55, -0.14] | 0%, 2.53, (P = 0.47) | Low^3,4^ |
| *(Sensitivity analysis ‘lower dose’*)* | *3 (43%)* | *337(56%)* | *SMD= -0.26 [-0.50, -0.02]* | *0%, 0.55, (P=0.76)* | - |
| *‘Higher dose’* | 3 (75%) | 338 (82%) | SMD=-0.53 [-0.78, -0.29] | 0%, 0.13, (P = 0.94) | Very low^1,3,4^ |
| **Positive symptoms** | **6 (86%)** | **952 (93%)** | **SMD=-0.42 [-0.56, -0.28]** | **0%, 7.67, (P = 0.57)** | **Low^3,4^** |
| *Sensitivity analysis** | *4 (67%)* | *825 (81%)* | *SMD= -0.40 [-0.56, -0.25]* | *0%, 5.15, (P=0.64)* | *-* |
| *Subgroup analysis:*  *‘Lower dose’* | 6 (86%) | 545 (90%) | SMD= -0.36 [-0.58, -0.14] | 22%; 6.44, (P = 0.27) | Low^3,4^ |
| *(Sensitivity analysis ‘lower dose’*)* | *4 (57%)* | *438 (73%)* | *SMD= -0.29 [-0.50, -0.08]* | *0%, 3.97, (P=0.41)* | *-* |
| *‘Higher dose’* | 4 (100%) | 407 (98%) | SMD=-0.49 [-0.72, -0.27] | 0%, 0.50, (P = 0.92) | Low^3,4^ |
| **Negative symptoms** | **6 (86%)** | **845 (83%)** | **SMD= -0.32 [-0.46, -0.18]** | **0%, 4.81, (P=0.85)** | **Low^3,4^** |
| *Sensitivity analysis** | *4 (67%)* | *825 (81%)* | *SMD= -0.30 [-0.45, -0.15]* | *0%, 3.41, (p=0.84)* | *-* |
| *Subgroup analysis:*  *‘Lower dose’* | 6 (86%) | 545 (90%) | SMD=-0.31 [-0.49, -0.12] | 0%, 2.44, (P = 0.79) | Low^3,4^ |
| *(Sensitivity analysis ‘lower dose’*)* | *5 (71%)* | *438 (73%)* | *SMD=-0.29 [-0.50, -0.08]* | *0%, 2.31, (P=0.68)* | *-* |
| *‘Higher dose’* | 4 (100%) | 407 (98%) | SMD=-0.34 [-0.56, -0.12] | 0%, 2.31, (P = 0.51) | Low^3,4^ |
| **Depression** | **3 (43%)** | **393 (39%)** | **SMD=-0.24 [-0.45, -0.03]** | **0%, 0.94, (P = 0.92)** | **Very low^1,3,4^** |
| *Sensitivity analysis** | *2 (29%)* | *373 (37%)* | *SMD=-0.22 [-0.44, -0.01]* | *0%, 0.30, (P=0.96)* | **-** |
| *Subgroup analysis:*  *‘Lower dose’* | 3 (43%) | 209 (35%) | SMD=-0.22 [-0.50, 0.07] | 0%, 0.85 (P = 0.65) | Very low^1,3,4^ |
| *‘Higher dose’* | 2 (50%) | 184 (44%) | SMD=-0.28 [-0.58, 0.03] | 0%, 0.01, (P = 0.92) | Very low^1,3,4^ |
| **Psychosocial functioning** | **4 (57%)** | **919 (90%)** | **SMD= -0.37 [-0.52, -0.23]** | **15%, 8.25, (P = 0.31)** | **Low^,3,4^** |
| *Subgroup analysis:*  *‘Lower dose*’ | 4 (57%) | 515 (85%) | SMD=-0.29 [-0.53, -0.05] | 43%, 5.29, (P = 0.15) | Low^,3,4^ |
| *‘Higher dose’* | 4 (100%) | 404 (98%) | SMD=-0.48 [-0.69, -0.27] | 0%, 1.13, (P = 0.77) | Low^3,4^ |
| **Global state severity** | **3 (43%)** | **621 (61%)** | **SMD= -0.41 [-0.58, -0.25]** | **0%, 1.79, (P = 0.77)** | **Low^3,4^** |
| *Sensitivity analysis** | *2 (29%)* | *514 (50%)* | *SMD= -0.37 [-0.55, -0.20]* | *0%, 0.65, (P=0.88)* | - |
| *Subgroup analysis:*  *‘Lower dose’* | 3 (43%) | 364 (60%) | SMD= -0.39 [-0.60, -0.18] | 0%, 1.56, (P = 0.46) | Very low^1,3,4^ |
| *(Sensitivity analysis ‘lower dose’*)* | *2 (29%)* | *257 (43%)* | *SMD= -0.32 [-0.56, -0.07]* | *0%, 0.02, (P=0.80)* | - |
| *‘Higher dose’* | 2 (50%) | 257 (62%) | SMD= -0.44 [-0.70, -0.18] | 0%, 0.14, (P = 0.71) | Very low^1,3,4^ |
| **Global state improvement** | **1 (14%)** | **221 (22%)** | **RR= 1.89 [1.26, 2.83]** | **NA** | **Very low^1,3,4^** |
| *Subgroup analysis:*  *‘Lower dose’* | 1 (14%) | 110 (18%) | RR= 1.82 [1.02, 3.25] | NA | Very low^1,3,4^ |
| *‘Higher dose’* | 1 (25%) | 111 (27%) | RR= 1.95 [1.10, 3.45] | NA | Very low^1,3,4^ |
| **Mean weight (kg)** | **4 (57%)** | **625 (61%)** | **SMD=0.63 [0.32, 0.93]** | **68%, 15.85,(P= 0.007)** | **Very Low^2,3,4^** |
| *Subgroup analysis:*  *‘Lower dose’* | 4 (57%) | 443 (73%) | SMD=0.65 [0.18, 1.13] | 81%, 15.74, (P=0.001) | Very low^2,3,4^ |
| *‘Higher dose’* | 2 (50%) | 182 (44%) | SMD=0.57 [0.26, 0.89] | 0%, 0.02, (P = 0.90) | Very low^1,3,4^ |
| **>7% weight (kg) gain***∞* | **2 (29%)** | **373 (37%)** | **RR=3.62 [1.29, 10.17]** | **0%, 1.22, (P=0.75)** | **Very low^1,3,4^** |
| *Subgroup analysis*  *‘Lower dose’* | 2 (29%) | 189 (31%) | RR=3.25 [0.68, 15.52] | 10%, 1.11 (P=0.29) | Very low^1,3,4^ |
| *‘Higher dose’* | 2 (57%) | 184 (44%) | RR=3.97 [0.94, 16.80] | 0%, 0.10 (p=0.75) | Very low^1,3,4^ |
| **Leaving the study early due to side effects** | **5 (71%)** | **942 (92%)** | **RR=2.44 [1.12, 5.31]** | **0%, 0.99, (P = 1.00)** | **Very Low^1,3,4^** |
| *Subgroup analysis:*  *‘Lower dose’* | 5 (71%) | 528 (87%) | RR=2.53 [0.87, 7.34] | 0%, 0.74, (P = 0.95) | Very low^1,3,4^ |
| *‘Higher dose’* | 4 (100%) | 414 (100%) | RR=2.33 [0.74, 7.30] | 0%, 0.24, (P= 0.97) | Low^1,3,4^ |
| **Sensitivity analysis was conducted where outcomes were measured using mean endpoint scores and mean change scores by different studies included in analysis.*  *∞ >7% of baseline weight*  FINDLING2012: ‘lower dose’=quetiapine 400mg/day, ‘higher dose’=quetiapine 800mg/day; post-treatment=26 weeks  FINDLING2008: ‘lower dose’=aripiprazole 10mg/day, ‘higher dose’=aripiprazole 30mg/day; post-treatment=6 weeks  HAAS2009B: ‘lower dose’=risperidone 1-3mg/day, ‘higher dose’=risperidone 4-6mg/day; post-treatment=6 weeks  KRYZHANOVSKAYA2009B: ‘lower dose’=olanzapine 11.1mg/day, ‘higher dose’=n/a; post-treatment=6 weeks  PALLIERE-MARTINOT1995: ‘lower dose’=50-100mg/day Amisulpride; post-treatment=6 weeks  SINGH2011: ‘lower dose’=paliperidone 1.5mg/day, ‘higher dose’=paliperidone 1-3mg/day; post-treatment=6 weeks | | | | | |

Risperidone versus olanzapine at post treatment

| **Outcome** | **Trials in analysis** | **Participants in analysis** | **Effect Estimate [95% CI],Random-effects** | **Heterogeneity:I^2^; Chi² (p value)** | **Quality** |
| --- | --- | --- | --- | --- | --- |
| **Total symptoms** | **5 (71%)** | **210 (36%)** | **SMD = 0.05 [-0.24, 0.33]** | **7%, 4.31, (P = 0.37)** | **Very low^1,3,4,5^** |
| *Subgroup analysis: FEP* | 3 (75%) | 150 (30%) | SMD = -0.09 [-0.41, 0.24] | 0%, 1.10, (P = 0.58) | Very low^1,3,4,5^ |
| *Acute* | 2 (67%) | 60 (74%) | SMD = 0.38 [-0.14, 0.89] | 0%, 0.93, (P = 0.33) | Very low^1,3,4^ |
| **Positive symptoms** | **5 (71%)** | **210 (36%)** | **SMD=-0.28 [-1.05, 0.48]** | **85%,27.23,(P<0.0001)** | **Very low^1,2,3,4,5^** |
| *Subgroup analysis: FEP* | 3 (75%) | 150 (30%) | SMD=-0.72 [-1.87, 0.43] | 90%,20.23,(P=<0.0001) | Very low^1,2,3,4,5^ |
| *Acute* | 2 (67%) | 60 (74%) | SMD=0.38 [-0.13, 0.89] | 0%, 0.23, (P = 0.63) | Very low^1,3,4^ |
| **Negative symptoms** | **5 (71%)** | **210 (36%)** | **SMD = 0.22 [-0.27, 0.71]** | **65%, 11.56, (P = 0.02)** | **Very low^1,2,3,4,5^** |
| *Subgroup analysis: FEP* | 3 (75%) | 150 (30%) | SMD = 0.22 [-0.53, 0.98] | 79%, 9.57, (P = 0.008) | Very low^1,2,3,4,5^ |
| *Acute* | 2 (67%) | 60 (74%) | SMD = 0.22 [-0.51, 0.96] | 50%, 1.99, (P = 0.16) | Very low^1,2,3,4^ |
| **Depression** | **2 (29%)** | **116 (20%)** | **SMD=-0.60 [-1.74, 0.53]** | **87%, 7.88, (P = 0.005)** | **Very low^1,2,3,4,5^** |
| **Psychosocial functioning** | **1 (14%)** | **25 (4%)** | **SMD = 0.25 [-0.54, 1.04]** | **N/A** | **Very low^1,3,4^** |
| **Global state severity** | **3 (43%)** | **143 (24%)** | **SMD = -0.01 [-0.34, 0.32]** | **0%, 0.52, (P = 0.07)** | **Very low^1,3,4,5^** |
| *Subgroup analysis: FEP* | 2 (50%) | 108 (21%) | SMD = -0.06 [-0.44, 0.32] | 0%, 0.23, (P = 0.63) | Very low^1,3,4,5^ |
| *Acute* | 1 (33%) | 35 (43%) | SMD = 0.15 [-0.52, 0.82] | N/A | Very low^1,3,4^ |
| **Mean weight (kg or lbs)** | **5 (71%)** | **199 (39%)** | **SMD = -0.34 [-0.77, 0.09]** | **53%, 8.51, (P = 0.07)** | **Very low^1,2,3,4,5^** |
| *Subgroup analysis: FEP* | 3 (75%) | 139 (27%) | SMD = -0.29 [-1.02, 0.45] | 76%, 8.39, (P = 0.02) | Very low^1,2,3,4,5^ |
| *Acute* | 2 (67%) | 60 (74%) | SMD = -0.36 [-0.87, 0.16] | 0%, 0.06, (P = 0.81) | Very low^1,3,4^ |
| **>7% weight (lbs) gain∞** | **2 (29%)** | **94 (16%)** | **RR=0.91 [0.47, 1.76]** | **73%, 3.65, (P=0.06)** | **Very low^1,2,3,4,5^** |
| *Subgroup analysis: FEP* | 1 (25%) | 74 (15%) | RR=0.68 [0.47, 0.98] | NA | Very low^1,3,4,5^ |
| *Acute* | 1 (33%) | 20 (25%) | RR=1.33 [0.74, 2.41] | NA | Very low^1,3,4^ |
| **Leaving the study early due to side effects** | **5 (71%)** | **423 (72%)** | **RR = 1.07 [0.67, 1.73]** | **0%, 2.86, (P = 0.58)** | **Very Low^1,3,4,5^** |
| *Subgroup analysis: FEP* | 2 (50%) | 342 (68%) | RR = 1.02 [0.62, 1.67] | 0%, 0.13, (P = 0.72) | Very low^1,3,4,5^ |
| *Acute* | 3 (100%) | 81 (100%) | RR = 2.02 [0.33, 12.26] | 7%, 2.15, (P = 0.34) | Very low^1,3,4^ |
| *∞ >7% of baseline weight*  JENSEN2008: post-treatment=12 weeks  MCEVOY2007: post-treatment=52 weeks  MOZES2006: post-treatment=12 weeks  SIKICH2004: post-treatment=8 weeks  SICKIH2008: post-treatment=52 weeks  VANBRUGGEN2003: post-treatment=6-10 weeks | | | | | |

Olanzapine versus quetiapine at post treatment

| **Outcome** | **Trials in analysis** | **Participants in analysis** | **Effect Estimate [95% CI],Random-effects** | **Heterogeneity:I^2^; Chi² (p value)** | **Quality** |
| --- | --- | --- | --- | --- | --- |
| **Total symptoms** | **2 (67%)** | **131 (39%)** | **SMD = -0.04 [-0.54, 0.46]** | **50%, 2.01, (P = 0.16)** | **Very low^1,2,3,4,5^** |
| **Positive symptoms** | **2 (67%)** | **131 (39%)** | **SMD = -0.42 [-0.77, -0.08]** | **0%, 0.76, (P = 0.38)** | **Very low^1,3,4,5^** |
| **Negative symptoms** | **2 (67%)** | **131 (41%)** | **SMD = 0.34 [-0.77, 1.45]** | **89%, 9.24, (P = 0.002)** | **Very low^1,2,3,4,5^** |
| **Depression** | **2 (67%)** | **131 (41%)** | **SMD=0.32 [-0.03, 0.66]** | **0%, 0.57, (P = 0.45)** | **Very low^1,3,4,5^** |
| **Psychosocial functioning** | **1 (33%)** | **50 (15%)** | **SMD = -0.35 [-0.91, 0.20]** | **NA** | **Very low^1,3,4^** |
| **Global state severity** | **2 (67%)** | **131 (39%)** | **SMD = 0.11 [-0.44, 0.66]** | **59%, 2.41, (P = 0.12)** | **Very low^1,2,3,4,5^** |
| **>7% weight (lbs) gain^∞^** | **3 (100%)** | **151 (45%)** | **RR = 1.86 [1.33, 2.61]** | **0%, 1.80, (P = 0.41)** | **Very low^1,3,4,5^** |
| *Subgroup analysis: FEP* | 2 (100%) | 131 (41%) | RR = 2.05 [1.41, 2.97] | 0%, 0.38, (P = 0.54) | Very low^1,3,4,5^ |
| *Acute* | 1 (100%) | 20 (100%) | RR = 1.20 [0.54, 2.67] | N/A | Very low^1,3,4^ |
| **Leaving the study early due to side effects** | **3 (100%)** | **337 (100%)** | **RR = 1.14 [0.57, 2.29]** | **0%, 0.40, (P = 0.53)** | **Very low^1,3,4,5^** |
| *Subgroup analysis: FEP* | 2 (100%) | 317 (100%) | RR = 1.09 [0.53, 2.22] | N/A | Very low^1,3,4,5^ |
| *Acute* | 1 (100%) | 20 (100%) | RR = 3.00 [0.14, 65.90] | N/A | Very low^1,3,4^ |
| **^∞^** >7% baseline weight (JENSEN2008; MCEVOY2007), weight gain not defined by ARANGO2009  ARANGO2009: post-treatment=26 weeks  JENSEN2008: post-treatment=12 weeks  MCEVOY2007: post-treatment=52 weeks | | | | | |

Olanzapine versus haloperidol at post treatment

| **Outcome** | **Trials in analysis** | **Participants in analysis** | **Effect Estimate [95% CI],Random-effects** | **Heterogeneity:I^2^; Chi² (p value)** | **Quality** |
| --- | --- | --- | --- | --- | --- |
| **Total symptoms** | **2 (100%)** | **282 (96%)** | **SMD = -0.32 [-0.70, 0.07]** | **30%, 1.43, (P = 0.23)** | **Very low^1,3,4,5^** |
| *Subgroup analysis: FEP* | 1 (100%) | 251 (95%) | SMD = -0.21 [-0.46, 0.04] | N/A | Very low^1,3,4,5^ |
| *Acute* | 1 (100%) | 31 (100%) | SMD = -0.68 [-1.41, 0.05] | N/A | Very low^1,3,4^ |
| **Positive symptoms** | **2 (100%)** | **283 (96%)** | **SMD = -0.20 [-0.69, 0.28]** | **48%, 1.92, (P = 0.17)** | **Very low^1,3,4,5^** |
| *Subgroup analysis: FEP* | 1 (100%) | 252 (96%) | SMD = -0.04 [-0.29, 0.20] | N/A | Very low^1,3,45^ |
| *Acute* | 1 (100%) | 31 (100%) | SMD = -0.58 [-1.30, 0.14] | N/A | Very low^1,3,4^ |
| **Negative symptoms** | **2 (100%)** | **283 (96%)** | **SMD = -0.22 [-0.46, 0.01]** | **0%, 0.43, (P = 0.51)** | **Very low^1,3,4,5^** |
| *Subgroup analysis: FEP* | 1 (100%) | 252 (96%) | SMD = -0.25 [-0.50, -0.00] | N/A | Very low^1,3,4,5^ |
| *Acute* | 1 (100%) | 31 (100%) | SMD = 0.00 [-0.70, 0.70] | N/A | Very low^1,3,4^ |
| **Global state severity** | **2 (100%)** | **285 (97%)** | **SMD = -0.32 [-0.79, 0.16]** | **47%, 1.88, (P = 0.17)** | **Very low^1,3,4,5^** |
| *Subgroup analysis: FEP* | 1 (100%) | 254 (97%) | SMD = -0.16 [-0.41, 0.08] | N/A | Very low^1,3,4,5^ |
| *Acute* | 1 (100%) | 31 (100%) | SMD = -0.70 [-1.43, 0.03] | N/A | Very low^1,3,4^ |
| **Mean weight (kg)** | **2 (100%)** | **294 (100%)** | **SMD = 0.38 [-0.38, 1.14]** | **76%, 4.25, (P = 0.04)** | **Very low^1,2,3,4,5^** |
| *Subgroup analysis: FEP* | 1 (100%) | 263 (100%) | SMD = 0.70 [0.45, 0.95] | N/A | Very low^1,3,4,5^ |
| *Acute* | 1 (100%) | 31 (100%) | SMD = -0.08 [-0.79, 0.62] | N/A | Very low^1,3,4^ |
| **Leaving the study early due to side effects** | **2 (100%)** | **294 (100%)** | **RR = 0.28 [0.08, 0.97]** | **21%, 1.27, (P = 0.26)** | **Very low^1,3,4,5^** |
| *Subgroup analysis: FEP* | 1 (100%) | 263 (100%) | RR = 0.37 [0.16, 0.85] | N/A | Very low^1,3,4,5^ |
| *Acute* | 1 (100%) | 31 (100%) | RR = 0.07 [0.00, 1.18] | N/A | Very low^1,3,4^ |
| LIEBERMAN2003: post-treatment 12 weeks  SIKICH2004: post-treatment 8 weeks | | | | | |

Risperidone versus haloperidol at post-treatment

| **Outcome** | **Trials in analysis** | **Participants in analysis** | **Effect Estimate [95% CI],Random-effects** | **Heterogeneity:I^2^; Chi² (p value)** | **Quality** |
| --- | --- | --- | --- | --- | --- |
| **Total symptoms** (*Acute*) | 2 (100%) | 76 (68%) | SMD = -0.33 [-0.79, 0.12] | 0%, 0.01, (P = 0.90) | Very low^1,3,4^ |
| **Positive symptoms** (*Acute*) | 1 (50%) | 34 (31%) | SMD = -0.25 [-0.93, 0.43] | N/A | Very low^1,3,4^ |
| **Negative symptoms** (*Acute)* | 1 (50%) | 34 (31%) | SMD = -0.11 [-0.79, 0.57] | N/A | Very low^1,3,4^ |
| **Global state severity** *(Acute)* | 1 (50%) | 34 (31%) | SMD = -0.54 [-1.23, 0.15] | N/A | Very low^1,3,4^ |
| **Mean weight (kg)** *(Acute)* | 1 (50%) | 34 (31%) | SMD = -0.40 [-1.09, 0.28] | N/A | Very low^1,3,4^ |
| **Leaving the study early due to side effects** | 1 (50%) | 35 (37%) | RR = 0.50 [0.17, 1.46] | N/A | Very low^1,3,4^ |
| * Data for FEP not available for risperidone versus haleperidol  KENNEDY2012/ YAO2003: post-treatment 6 weeks  SIKICH2008b: post-treatment 52 weeks | | | | | |

Risperidone versus quetiapine at post treatment

| **Outcome** | **Trials in analysis** | **Participants in analysis** | **Effect Estimate [95% CI],Random-effects** | **Heterogeneity:I^2^; Chi² (p value)** | **Quality** |
| --- | --- | --- | --- | --- | --- |
| **Total symptoms** | **2 (67%)** | **103 (33%)** | SMD=-0.28 [-0.67, 0.11] | 0%, 0.00, (P = 0.98) | **Very low^1,3,4,5^** |
| **Positive symptoms** | **2 (67%)** | **103 (33%)** | SMD=-0.43 [-0.82, -0.03] | 0%, 0.11, (P = 0.74) | **Very low^1,3,4,5^** |
| **Negative symptoms** | **2 (67%)** | **103 (33%)** | SMD=-0.22 [-0.61, 0.17] | 0%, 0.03, (P = 0.86) | **Very low^1,3,4,5^** |
| **Depression** | **1 (33%)** | **81 (26%)** | **SMD=0.38 [-0.07, 0.82]** | **N/A** | **Very low^1,3,4,5^** |
| **Global state** | **2(67%)** | **103 (33%)** | SMD=-0.14 [-0.53, 0.25] | 0%, 0.00, (P = 0.97) | **Very low^1,3,4,5^** |
| **Mean weight (kg or lbs)** | **2 (67%)** | **103 (33%)** | SMD=0.13 [-0.26, 0.52] | 0%, 0.25, (P = 0.62) | **Very low^1,3,4,5^** |
| **>7% weight (kg or lbs) gain**∞ | **3 (100%)** | **123 (40%)** | **RR=1.45 [0.95, 2.21]** | 3%, 2.07 (P=0.36) | **Very low^1,3,4,5^** |
| *Subgroup analysis: FEP* | 2 (100%) | 103 (36%) | RR=1.00 [0.26, 3.81] | 48%, 1.93 (P=0.17) | Very low^1,3,4,5^ |
| *Acute* | 1 (100%) | 20 (100%) | RR=1.60 [0.80, 3.20] | NA | Very low^1,3,4^ |
| **Leaving the study early due to side effects** | **3 (100%)** | **309 (100%)** | RR= 1.01 [0.49, 2.09] | **NA**^∞^ | **Very low^1,3,4,5^** |
| *Subgroup analysis: FEP* | 2 (100%) | 289 (100%) | RR=1.01 [0.49, 2.09] | NA^∞^ | Very low^1,3,4,5^ |
| *Acute* | 1 (100%) | 20 (100%) | Not estimable± | N/A | Very low^1,3,4^ |
| ∞*>7% of baseline weight*  ± No events observed in the SWADI2012 or JENSEN2008 trials  JENSEN2008: post-treatment 6 weeks  MCEVOY2007: post-treatment 52 weeks | | | | | |

Risperidone versus chlorpromazine at post treatment

| **Outcome** | **Trials in analysis** | **Participants in analysis** | **Effect Estimate [95% CI],**  **Random-effects** | **Heterogeneity:**  **I^2^; Chi² (p value)** | **Quality** |
| --- | --- | --- | --- | --- | --- |
| **Total symptoms** | 1 (100%) | 60 (100%) | SMD = -0.29 [-0.80, 0.22] | N/A | Very low^1,3,4^ |
| KENNEDY2012/XIONG2003: post-treatment 8 weeks | | | | | |

| *Note to pharmacological interventions.*  *For each intervention the following outcomes were extracted if reported in the study: total symptoms, positive symptoms, negative symptoms, psychosocial functioning, weight, leaving the study early due to side effects.*  **Sensitivity analysis was conducted when mean endpoint scores and mean change scores were used within one analysis.*  *FEP=first episode psychosis; Acute=subsequent acute episode; SMD=standard mean difference; RR=risk ratio; CI=confidence interval*  *Reasons for downgrading:*  *^1^imprecision (optimal information size for dichotomous outcomes=300 events, and for continuous outcomes=400 participants).*  *^2^inconsistency (I^2^>50%, p<0.10)*  *^3^risk of bias (selection bias, performance bias, detection bias and/or attrition bias, selective outcome reporting bias)*  *^4^risk of publication bias*  *^5^indirectness (RCT(s) meets eligibility criteria but address a restricted version of the main review question in terms of population. MCEVOY2007: upper age range 44 years; LIEBERMAN2003: upper age range 40years; SCHOOLER2005: 48% population bipolar disorder).* |
| --- |

**Psychological Interventions**

Individual CBT plus family CBT versus TAU (EPPIC) at post-treatment (33 weeks)

| **Outcome** | **Trials in analysis** | **Participants in analysis** | **Effect Estimate (95% CI), Random-effects** | **Heterogeneity: I^2^; Chi² (p value)** | **Quality** |
| --- | --- | --- | --- | --- | --- |
| **Total symptoms** | 1 (100%) | 81 (99%) | SMD = -0.08 [-0.51, 0.36] | N/A | Low^1,3^ |
| **Positive symptoms** | 1 (100%) | 81 (99%) | SMD = -0.28 [-0.72, 0.15] | N/A | Low^1,3^ |
| **Negative symptoms** | 1 (100%) | 81 (99%) | SMD = -0.03 [-0.46, 0.41] | N/A | Low^1,3^ |
| **Relapse (Time in Days)** | 1 (100%) | 81 (99%) | SMD = -3.26 [-3.94, -2.59] | N/A | Low^1,3^ |
| **Relapse (Number of Participants)** | 1 (100%) | 81 (99%) | RR = 0.24 [0.06, 1.08] | N/A | Low^1,3^ |
| **Depression** | 1 (100%) | 81 (99%) | SMD = -0.24 [-0.68, 0.20] | N/A | Low^1,3^ |
| **Psychosocial functioning** | 1(100%) | 81 (99%) | SMD= 0.06 [-0.37, 0.50] | N/A | Low^1,3^ |
| **Leaving the study early for any reason** | 1 (100%) | 82 (100%) | RR = 1.40 [0.48, 4.05] | N/A | Low^1,3^ |

Individual CBT plus family CBT versus TAU (EPPIC) at follow-up (130 weeks)

| **Outcome** | **Trials in analysis** | **Participants in analysis** | **Effect Estimate (95% CI), Random-effects** | **Heterogeneity: I^2^; Chi² (p value)** | **Quality** |
| --- | --- | --- | --- | --- | --- |
| **Total symptoms** | 1 (100%) | 81 (99%) | SMD = 0.17 [-0.27, 0.60] | N/A | Low^1,3^ |
| **Positive symptoms** | 1 (100%) | 81 (99%) | SMD = -0.07 [-0.50, 0.37] | N/A | Low^1,3^ |
| **Negative symptoms** | 1 (100%) | 81 (99%) | SMD =0.60 [0.15, 1.05] | N/A | Low^1,3^ |
| **Relapse (Number of Participants)** | 1 (100%) | 81 (99%) | RR = 0.98 [0.31, 3.11] | N/A | Low^1,3^ |
| **Depression** | 1 (100%) | 81 (99%) | SMD = 0.02 [-0.42, 0.45] | N/A | Low^1,3^ |
| **Psychosocial functioning** | 1(100%) | 81 (99%) | SMD= -0.45 [-0.89, -0.01] | N/A | Low^1,3^ |
| **Leaving the study early for any reason** | 1 (100%) | 82 (100%) | RR = 1.10 [0.53, 2.30] | N/A | Low^1,3^ |

Individual CBT versus TAU (in the UK) at post treatment (24 weeks)

| **Outcome** | **Trials in analysis** | **Participants in analysis** | **Effect Estimate (95% CI),**  **Random-effects** | **Heterogeneity:**  **I2; Chi² (p value)** | **Quality** |
| --- | --- | --- | --- | --- | --- |
| **Depression** | 1 (50%) | 46 (17%) | SMD = -0.29 [-0.87, 0.30] | N/A | Low^1,3^ |
| **Leaving the study early for any reason** | 1 (50%) | 66 (25%) | SMD = 1.94 [0.85, 4.43] | N/A | Low^1,3^ |

Individual CBT versus TAU (in the UK) at follow-up (52 weeks)

| **Outcome** | **Trials in analysis** | **Participants in analysis** | **Effect Estimate (95% CI), Random-effects** | **Heterogeneity: I^2^; Chi² (p value)** | **Quality** |
| --- | --- | --- | --- | --- | --- |
| **Depression** | 1 (50%) | 46 (17%) | SMD = -0.05 [-0.63, 0.52] | N/A | Low^1,3^ |
| **Leaving the study early for any reason** | 1 (50%) | 66 (25%) | RR = 1.77 [0.89, 3.52] | N/A | Low^1,3^ |

Individual CBT plus TAU (EPPIC) versus befriending at post treatment (12 weeks)

| **Outcome** | **Trials in analysis** | **Participants in analysis** | **Effect Estimate (95% CI), Random-effects** | **Heterogeneity: I^2^; Chi² (p value)** | **Quality** |
| --- | --- | --- | --- | --- | --- |
| **Positive symptoms** | 1 (100%) | 62 (100%) | SMD = -0.05 [-0.55, 0.45] | N/A | Very low^1,3,5^ |
| **Negative symptoms** | 1 (100%) | 62 (100%) | SMD = -0.44 [-0.95, 0.06] | N/A | Very low^1,3,5^ |
| **Psychosocial functioning** | 1 (100%) | 62 (100%) | SMD=-0.40 [-0.90, 0.11] | N/A | Very low^1,3,5^ |
| **Leaving the study early for any reason** | 1 (100%) | 62 (100%) | RR = 0.57 [0.19, 1.76] | N/A | Very low^1,3,5^ |

Individual CBT plus TAU (EPPIC) versus befriending at follow-up (52 weeks)

| **Outcome** | **Trials in analysis** | **Participants in analysis** | **Effect Estimate (95% CI), Random-effects** | **Heterogeneity: I^2^; Chi² (p value)** | **Quality** |
| --- | --- | --- | --- | --- | --- |
| **Positive symptoms** | 1 (100%) | 62 (100%) | SMD = -0.08 [-0.58, 0.42] | N/A | Very low^1,3,5^ |
| **Negative symptoms** | 1 (100%) | 62 (100%) | SMD = -0.37 [-0.87, 0.13] | N/A | Very low^1,3,5^ |
| **Psychosocial functioning** | 1 (100%) | 62 (100%) | SMD= -0.08 [-0.58, 0.41] | N/A | Very low^1,3,5^ |

Individual CBT versus TAU (EPPIC) in acutely suicidal participants at post-treatment (10 weeks)

| **Outcome** | **Trials in analysis** | **Participants in analysis** | **Effect Estimate (95% CI), Random-effects** | **Heterogeneity: I^2^; Chi² (p value)** | **Quality** |
| --- | --- | --- | --- | --- | --- |
| **Leaving the study early for any reason** | 1 (100%) | 56 (100%) | RR = 2.02 [0.72, 5.66] | N/A | Very low^1,3,5^ |

Family CBT versus individual CBT at post-treatment (66 weeks)

| **Outcome** | **Trials in analysis** | **Participants in analysis** | **Effect Estimate (95% CI),**  **Random-effects** | **Heterogeneity:**  **I^2^; Chi² (p value)** | **Quality** |
| --- | --- | --- | --- | --- | --- |
| **Relapse** | 1 (100%) | 76 (100%) | RR=0.95 [0.34, 2.68] | N/A | Low^1,3^ |

| *Note to psychological interventions.*  *For each intervention the following outcomes were extracted if reported in the study: total symptoms, positive symptoms, negative symptoms, psychosocial functioning, weight, leaving the study early due to side effects.*  *SMD=standard mean difference; RR=risk ratio; CI=confidence interval*  *Reasons for downgrading:*  *^1^imprecision (optimal information size for dichotomous outcomes=300 events, and for continuous outcomes=400 participants)*  *^2^inconsistency (I^2^>50%, p<0.10)*  *^3^risk of bias (selection bias, performance bias, detection bias and/or attrition bias)*  *^4^risk of publication bias (selective outcome reporting)*  *^5^indirectness (RCT(s) meets eligibility criteria but address a restricted version of the main review question in terms of population. JACKSON2008: 21% of participants had bipolar and 8.1% of participants were receiving ECT; POWER2003: participants acutely suicidal)* |
| --- |

Appendix I: PRISMA checklist

| **Section/topic** | **#** | **Checklist item** | **Reported on page #** |
| --- | --- | --- | --- |
| **TITLE** | | |  |
| Title | 1 | Identify the report as a systematic review, meta-analysis, or both. | 1 |
| **ABSTRACT** | | |  |
| Structured summary | 2 | Provide a structured summary including, as applicable: background; objectives; data sources; study eligibility criteria, participants, and interventions; study appraisal and synthesis methods; results; limitations; conclusions and implications of key findings; systematic review registration number. | 2 |
| **INTRODUCTION** | | |  |
| Rationale | 3 | Describe the rationale for the review in the context of what is already known. | 4-5 |
| Objectives | 4 | Provide an explicit statement of questions being addressed with reference to participants, interventions, comparisons, outcomes, and study design (PICOS). | 5 |
| **METHODS** | | |  |
| Protocol and registration | 5 | Indicate if a review protocol exists, if and where it can be accessed (e.g., Web address), and, if available, provide registration information including registration number. | 5 |
| Eligibility criteria | 6 | Specify study characteristics (e.g., PICOS, length of follow-up) and report characteristics (e.g., years considered, language, publication status) used as criteria for eligibility, giving rationale. | 5-6 |
| Information sources | 7 | Describe all information sources (e.g., databases with dates of coverage, contact with study authors to identify additional studies) in the search and date last searched. | 6 |
| Search | 8 | Present full electronic search strategy for at least one database, including any limits used, such that it could be repeated. | Appendix S2 |
| Study selection | 9 | State the process for selecting studies (i.e., screening, eligibility, included in systematic review, and, if applicable, included in the meta-analysis). | 5-6 |
| Data collection process | 10 | Describe method of data extraction from reports (e.g., piloted forms, independently, in duplicate) and any processes for obtaining and confirming data from investigators. | 7-8 |
| Data items | 11 | List and define all variables for which data were sought (e.g., PICOS, funding sources) and any assumptions and simplifications made. | 5 |
| Risk of bias in individual studies | 12 | Describe methods used for assessing risk of bias of individual studies (including specification of whether this was done at the study or outcome level), and how this information is to be used in any data synthesis. | 6-7 |
| Summary measures | 13 | State the principal summary measures (e.g., risk ratio, difference in means). | 7 |
| Synthesis of results | 14 | Describe the methods of handling data and combining results of studies, if done, including measures of consistency (e.g., I^2^) for each meta-analysis. | 8 |
